# PKCθ-JunB axis via upregulation of VEGFR3 expression mediates hypoxia-induced pathological retinal neovascularization

**DOI:** 10.1038/s41419-020-2522-0

**Published:** 2020-05-07

**Authors:** Raj Kumar, Arul M. Mani, Nikhlesh K. Singh, Gadiparthi N. Rao

**Affiliations:** Department of Physiology, University of Tennessee Health Science Center, Memphis, TN 38163 USA

**Keywords:** Biochemistry, Diseases

## Abstract

Pathological retinal neovascularization is the most common cause of vision loss. PKCθ has been shown to play a role in type 2 diabetes, which is linked to retinal neovascularization. Based on these clues, we have studied the role of PKCθ and its downstream target genes JunB and VEGFR3 in retinal neovascularization using global and tissue-specific knockout mouse models along with molecular biological approaches. Here, we show that vascular endothelial growth factor A (VEGFA) induces PKCθ phosphorylation in human retinal microvascular endothelial cells (HRMVECs) and downregulation of its levels attenuates VEGFA-induced HRMVECs migration, sprouting and tube formation. Furthermore, the whole body deletion of PKCθ or EC-specific deletion of its target gene JunB inhibited hypoxia-induced retinal EC proliferation, tip cell formation and neovascularization. VEGFA also induced VEGFR3 expression via JunB downstream to PKCθ in the regulation of HRMVEC migration, sprouting, and tube formation in vitro and OIR-induced retinal EC proliferation, tip cell formation and neovascularization in vivo. In addition, VEGFA-induced VEGFR3 expression requires VEGFR2 activation upstream to PKCθ-JunB axis both in vitro and in vivo. Depletion of VEGFR2 or VEGFR3 levels attenuated VEGFA-induced HRMVEC migration, sprouting and tube formation in vitro and retinal neovascularization in vivo and it appears that these events were dependent on STAT3 activation. Furthermore, the observations using soluble VEGFR3 indicate that VEGFR3 mediates its effects on retinal neovascularization in a ligand dependent and independent manner downstream to VEGFR2. Together, these observations suggest that PKCθ-dependent JunB-mediated VEGFR3 expression targeting STAT3 activation is required for VEGFA/VEGFR2-induced retinal neovascularization.

## Introduction

Retinal neovascularization is the most common cause of vision loss in various ocular diseases such as retinopathy of prematurity (ROP), diabetic retinopathy (DR) and age-related macular degeneration (AMD)^1–3^. Vascular endothelial growth factor-A (VEGFA) appears to be a major player in these ocular diseases, as anti-VEGFA therapies have been shown to be effective in the treatment of these diseases^4,5^. VEGFA binds to both VEGFR1 and VEGFR2, although the latter appears to mediate most of its biological effects^6^. It was shown that interactions between VEGFR2 and VEGFR3 are required for sprouting angiogenesis as well as blood vessel development^7,8^. On the other hand, it was reported that VEGFR3, which preferentially binds its ligands VEGFC and VEGFD, alone is required for developmental angiogenesis, as its knockdown results in embryonic lethality^9^. Furthermore, it was demonstrated that VEGFR3 is expressed highly in tip cells and promotes angiogenesis and vessel growth even in the absence of VEGFR2 activation^10^. Besides, in the absence of VEGFA-VEGFR2 signaling and low Notch activity, ligand-independent increase in VEGFR3 kinase activity has been shown to be sufficient in the modulation of retinal angiogenesis^10^. Contrary to these observations, it has also been shown that VEGFR3 promotes tip to stalk cell conversion via augmenting Notch signaling and its genetic inactivation results in excessive sprouting and vessel branching^11^. In addition, the studies by Zarkada et al. have reported that VEGFR2 mediates postnatal sprouting angiogenesis independent of the involvement of VEGFR3^12^. This study has further shown that VEGFR3 fails to induce sprouting angiogenesis even in the presence of low Notch signaling. However, recently, it was reported that genetic deletion of VEGFR3 leads to increased VEGFR2 expression resulting in heightened vascular permeability^13^. Despite all these studies on the controversial crosstalk between VEGFR2 and VEGFR3 in the modulation of postnatal sprouting angiogenesis or developmental angiogenesis, our understanding about their interactions in the modulation of pathological angiogenesis is unknown.

Many studies have identified the importance of diacylglycerol (DAG), an endogenous protein kinase C (PKC) activator, in hyperglycemia-induced structural and functional abnormalities in vascular tissues^14^. VEGFA via binding to VEGFR2 triggers phosphoinositol hydrolysis generating DAG, which in turn, leads to activation of novel PKCs^15^. A study utilizing PKCθ knockout mice model has demonstrated that genetic deletion of PKCθ protects mice from fat-induced insulin resistance and suggested that PKCθ could be a potential target for the treatment of diabetes^16^. Furthermore, it was reported that activator protein 1 (AP1) participates in the signal transduction of PKC-triggered cell proliferation^17^. In addition, we have shown that c-Jun and Fra1 induction is required for adaptative angiogenesis in a hindlimb ischemia model^18^. Based on these clues, we asked the question whether PKCθ via AP1 plays a role in pathological retinal neovascularization, and if so, the relative involvement of VEGFR2 and VEGFR3. Our results for the first time show that PKCθ-JunB axis mediates VEGFR3 expression and is required for VEGFA/VEGFR2-induced angiogenic events in human retinal microvascular endothelial cells and OIR-induced mouse retinal neovascularization.

## Materials And Methods

### Reagents

Recombinant human VEGF165a (Cat. No. 293-VE-010/CF), recombinant mouse VEGF164a (Cat. No. 493-MV-025/CF), recombinant VEGFR2 Fc Chimera protein (Cat. No. 357-KD-050/CF) and recombinant VEGFR3 Fc Chimera protein (Cat. No. 349-F4-50/CF) were bought from R&D Systems (Minneapolis, MN). Growth factor-reduced Matrigel (Cat. No. 354230) was purchased from BD Biosciences (Bedford, MA). Anti-cFos (SC-52), anti-cJun (Sc-1694 and SC-7454×), anti-Fra1 (SC-183), anti-Fra2 (SC-13017), anti-JunB (SC-73), anti-JunD (SC-74 and SC-271938×), normal mouse IgG (SC-2025), normal rabbit IgG (SC-2027), anti-PKCθ (SC-212), anti-STAT3 (SC-482), anti-VEGFR3 (SC-321 and SC-514825) anti-α-Tubulin (SC-23948) and anti-β-Tubulin (SC-91044) antibodies were obtained from Santa Cruz Biotechnology (Santa Cruz, CA). Anti-JunB (Cat. No. 3753), anti-pPKCθ (Cat. No. 9377), anti-pSTAT3 (Cat. No. 9131), anti-pVEGFR2 (Cat. No. 2478) and anti-VEGFR2 (Cat. No. 2479) antibodies were purchased from Cell Signaling Technology (Beverly, MA). Anti-CD31 (Cat. No. 550274) was bought from BD Pharmingen (Palo Alto, CA). Anti-Ki67 (ab15580), and anti-VEGFR1 (ab32152) were obtained from Abcam (Cambridge, MA). Anti-pTyr (PY20) (Cat. No. 05-777) was obtained from EMD Millipore (Burlington, MA). Human PKCθ siRNA (Set-1) (1429936), human PKCθ siRNA (Set-2) (s11122), mouse PKCθ siRNA (s71711), human JunB siRNA (Set-1) (115275), human JunB siRNA (Set-2) (s223956), human VEGFR3 siRNA (Set-1) (145459), human VEGFR3 siRNA (Set-2) (s5294), human VEGFR2 siRNA (s535305), mouse VEGFR2 siRNA (s68715) and human STAT3 siRNA (s743) were obtained from Thermo Scientific (Chicago, IL). Control non-targeting siRNA (D-001810-10), human PKCθ siRNA (Set-3) (L-003525-00), human JunB siRNA (Set-3) (L-003269-00), mouse JunB siRNA (L-041158-00) and mouse VEGFR3 siRNA (L-045433-00) were obtained from Dharmacon (Pittsburgh, PA). [^3^H]-Thymidine (S.A. 20 Ci/mmole) was bought from Perkin Elmer (Boston, MA). Endothelial cell basal medium (EBM) (CC-3133), endothelial cell growth medium (EGM) SingleQuot kit supplements and growth factors (CC-4133) were purchased from Lonza (Walkersville, MD). VEGFR3 inhibitor (MAZ51) (Cat. No. 676492) was obtained from EMD Millipore (USA). Cytodex microcarrier bead (Cat. No. C3278) and thrombin (Cat. No. T8885) were purchased from Sigma Aldrich (St Louis, MO). Fibrinogen (Cat. No. 820224) was obtained from MP Biomedicals LCC (Solon, OH). Biotin 3ʹ-end DNA labeling kit (Cat. No. 89818), Dyna beads (Cat. No. 11035), invivofectamine 3.0 (Cat. No. IVF3001), LightShift chemiluminescent EMSA kit (Cat. No. 20148) and lipofectamine 3000 transfection reagent (Cat. No. L-3000-015) were purchased from Thermo Fisher Scientific (Waltham, MA). Isolectin B4-594 (I21413), cell tracker green (C7025), goat anti-rabbit IgG-AlexaFluor 488 (A11034), goat anti-rat IgG-AlexaFluor 568 (A11077), goat anti-rat IgG-AlexaFluor 350 (A21093), Hoechst 3342, Prolong Gold antifade reagent were bought from Molecular Probes (Eugene, OR). Mouse JunB overexpression plasmid (Cat. No. MR336772) was obtained from Origene Technologies Inc. (Rockville, MD).

### Animals

JunB^flox/flox^ mice (stock # 012369) and PKCθ^−/−^ mice (stock # 005711) were purchased from Jackson Laboratories, Bar Harbor, ME. Tamoxifen-inducible recombinase (CreERT2) under the control of the endothelial Cdh5 promoter (Cdh5-Cre^ERT2^) mice were obtained from Dr. Luisa Iruela-Arispe, University of California at Las Angles, CA. To delete JunB in the postnatal vasculature, we interbred JunB^flox/flox^ mice with Cdh5-Cre^ERT2^ mice. JunB^flox/-^:Cdh5-Cre^ERT2^ mice were backcrossed with JunB^flox/flox^ to generate JunB^flox/flox^:Cdh5-Cre^ERT2^ litters. To induce Cre activity and gene deletion, two consecutive intraperitoneal tamoxifen (Sigma-Aldrich, T5648; 2 mg/ml; generated by dissolving in 10% ethanol and 90% corn oil) injections of 50 μl were given at P10 and P11 to generate endothelial specific deletion of JunB (JunB^iΔEC^). C57BL/6 (WT) pregnant mice (Strain code 027) at E16 were obtained from Charles River Laboratories, Wilmington, MA. Mice were bred and maintained at the University of Tennessee Health Science Center’s vivarium. All the experiments involving animals were approved by Institutional Animal Care and Use Committee (IACUC) of the University of Tennessee Health Science Center, Memphis, TN.

### Isolation of mouse retinal microvascular endothelial cells (MRMVECs)

Sheep anti-rat Dynabeads (Cat. No. 11035, Life Technologies, Grand Island, NY) were washed three times with serum-free Dulbecco’s modified Eagle’s medium (DMEM) and then incubated with rat anti-mouse CD31 antibody (Cat. No. 550330, BD Biosciences, San Jose, CA) for overnight at 4 °C (10 μl antibody/50 μl beads in DMEM). Eyes from 3-week-old WT or PKCθ^−/−^ mice pups were enucleated and retinas were dissected out and kept in phosphate-buffered saline (PBS) containing penicillin/streptomycin. Retinas were pooled, rinsed with PBS, minced into small pieces using sterilized razor blade and digested in 4 ml of collagenase type I (1 mg/ml in serum-free DMEM, Cat. No. LS004196, Worthington, Lakewood, NJ) for 60 min at 37 °C. Following digestion, DMEM with 10% fetal bovine serum (FBS) was added to the cellular digest, filtered through a sterile 40-μm nylon mesh (Cat. No. 352340, BD Biosciences, San Jose, CA) and centrifuged at 1000 rpm for 5 min to pellet the cells. After washing with DMEM containing 10% FBS twice, the cell pellet was resuspended in 1.0 ml DMEM with 10% FBS and incubated with sheep anti-rat magnetic beads pre-coated with anti-CD31 antibody. After affinity binding of cells, magnetic beads were washed six times with DMEM containing 10% FBS and the bound cells were plated onto a single well of a 24-well plate. Endothelial cells were grown in EGM-2 medium containing 20% FBS. Cells were maintained at 37 °C with 5% CO_2_ atmosphere and propagated in 1% gelatin-coated dishes.

### Oxygen-induced retinopathy (OIR)

OIR was performed as per the method of Smith et al.^19^ and quantified as described by Singh et al.^20^ C57BL/6, JunB^flox/flox^:Cdh5-Cre^ERT2^ and PKCθ^−/−^ mice pups at P7 along with their dams were exposed to 75% oxygen for 5 days and then returned to room air at P12. Mice pups of the same age kept at room air were used as controls. All the pups were sacrificed at P17 and eyes were enucleated and fixed in 4% (w/v) paraformaldehyde for 1 h at room temperature. Retinas were isolated, stained with isolectin B4, flat mounts were made, placed under a coverslip and examined under a Zeiss inverted fluorescence microscope (Axiovision Observer.z1). Retinal neovascularization was quantified by first setting a scale with a tolerance point of 50 based on the fluorescence intensity in the screenshot using a Nikon NIS-Elements software version AR3.1. Neovascularity was highlighted in red and then quantified by dividing the fluorescence intensity in the highlighted area by the total fluorescence intensity in the screenshot (*n* = 6 eyes).

### Intravitreal injections

Small interference RNA (siRNA) solutions were prepared by mixing equal volume of siRNA duplex (at 4 mg/ml) with equal volume of complexation buffer, which was then mixed with an equal volume of invivofectamine 2 transfection reagent (Thermo Fisher). The invivofectamine 2-siRNA duplex mixtures were then incubated at 50 °C for 30 min followed by dilution (6×) with PBS. The resulting mixture was concentrated using Amicon Ultra-15 Ultracel-50K filters to achieve the final concentration of siRNA to 2.0 mg/ml. Non-targeted or on-targeted siRNA at 1 µg/0.5 µl/eye was injected into mice pups at the indicated time periods of hyperoxia or hypoxia by intravitreal injections using a 33G needle. Soluble VEGFR2 or VEGFR3 dissolved in sterile PBS were injected intravitreally at 0.05 µg/0.5 µl/eye. Ad-GFP or Ad-dnSTAT3 were injected intravitreally at 40 moi/0.5 µl/eye.

### Cell culture

HRMVECs (Cat. No. ACBRI 181) were purchased from Cell Systems (Kirkland, WA) and grown in EGM containing growth supplements, 10 µg/ml gentamycin, and 0.25 µg/ml amphotericin B. Human fibroblasts (Cat. No. PCS-201-013) were obtained from American Type Culture Collection (Manassas, VA) grown in EGM containing growth supplements, 10 µg/ml gentamycin, and 0.25 µg/ml amphotericin B. Cultures were maintained at 37 °C in a humidified 95% air and 5% CO_2_ atmosphere. HRMVECs with passage numbers between 5 and 10 were synchronized by maintaining in growth factor-free EBM for 24 h and used to perform experiments unless otherwise indicated.

### Cell migration

Cell migration was performed using a modified Boyden chamber method^21^. The cell culture inserts (Cat. No. PI8P01250, Millipore) containing porous membrane of 12 mm in diameter and 8.0 μm pore size were first coated with growth factor reduced matrigel (Cat. No. 354230, Corning) on the lower surface of the membrane and kept at room temperature for 2 h to dry. The cell culture inserts were then placed in a 24-well tissue culture plate. HRMVECs or MRMVECs were quiesced for 24 h in serum-free EBM2, washed with PBS, trypsinized, and pelleted by centrifugation. Cells were resuspended in serum-free EBM2 and seeded into the upper chamber at 1 × 10^5^ cells/well. Wherever siRNA or plasmids were used, cells were transfected with non-targeted or on-targeted siRNA or pCMV empty vector or the indicated expression vector or infected with Ad-GFP or Ad-dnSTAT3 were allowed to grow to 70-80% confluence, synchronized for 24 h and then plated onto the upper chamber. Vehicle or VEGFA (40 ng/ml) were added to the lower chamber. After 12 h of incubation at 37 °C, non-migrated cells were removed from the upper side of the membrane with a cotton swab and the membrane was fixed in methanol and mounted on a slide with ProLong Gold mounting medium containing DAPI (4′,6-diamidino-2-phenylindole). The DAPI-positive cells were counted under an inverted fluorescence microscope (Zeiss Axio Observer.z1) and the cell migration was expressed as the number of cells per microscopic field.

### DNA synthesis

DNA synthesis was measured by [^3^H]-thymidine incorporation. Briefly, HRMVECs and MRMVECs transfected with non-targeted or on-targeted siRNA or infected with Ad-GFP or Ad-dnSTAT3 were plated onto 6-well plates, allowed to grow to 70-80% confluence, synchronized for 24 h and then treated with or without VEGFA (40 ng/ml) for 24 h. After 6 h of VEGFA addition, the cells were pulse-labeled with 1 µCi/ml of [^3^H]-thymidine for 18 h. After 24 h incubation period, cells were washed with cold PBS, trypsinized and pelleted by centrifugation. The cell pellet was resuspended in 3 ml of 20% (w/v) cold TCA and vortexed vigorously to lyse cells. The mixture was incubated on ice for 30 min and then passed through a GF/F glass microfiber filter. The filter was washed first with 3 ml of 5% cold TCA and then 3 ml of cold ethanol, dried, placed in liquid scintillation vial containing 5 ml of scintillation fluid and the radioactivity was counted in liquid scintillation counter (Beckman LS 3801). DNA synthesis was expressed as counts/min/well or fold changes.

### Three-dimensional (3D) Sprouting assay

Three-dimensional sprouting assay was performed as described earlier^22^. Briefly, HRMVECs transfected with non-targeted or on-targeted siRNA or MRMVECs transfected with pCMV empty vector or JunB expression vector or infected with Ad-GFP or Ad-dnSTAT3 were labeled with cell tracker, trypsinized, pelleted and equal number of cells were coated onto Cytodex beads for 6 h. The non-binding cells were washed with PBS and the beads were embedded in fibrin gel. Human fibroblasts suspended in EGM containing with and without VEGFA (40 ng/ml) were seeded on the top of fibrin gel at a concentration of 2 ×10^4^ cells/well and incubated at 37 °C for 6 h, at which time, medium was replaced with fresh EGM with and without VEGFA and incubation continued for the indicated time periods. Sprouting was examined on day 3 under Zeiss inverted fluorescence microscope (AxioVision Observer.z1; 10×/NA 0.45) and the fluorescence images were captured using Zeiss AxioCam MRm camera and the microscope operating and image analysis software AxioVision 4.7.2 (Carl Zeiss Imaging Solutions GmbH). Sprouting was expressed as number of sprouts per bead.

### Tube formation

Tube formation was measured as described earlier^20^. HRMVECs transfected with non-targeted or on-targeted siRNA or MRMVECs transfected with pCMV empty vector or JunB expression vector or infected with Ad-GFP or Ad-dnSTAT3 were synchronized for 24 h and plated in 24-well culture plate coated with growth factor-reduced Matrigel (BD Biosciences). Cells were added with or without VEGFA (40 ng/ml) and incubation continued for 6 h at 37 °C. Tube formation observed under an inverted phase contrast microscope (Eclipse TS100; Nikon, Tokyo, Japan) and the images were captured with CCD color camera (KP-D20AU; Hitachi, Ibaraki, Japan) using Apple iMovie 7.1.4 software. Tube length was calculated using NIH ImageJ version 1.43 and expressed in micrometers.

### RT-PCR

Total cellular RNA was extracted from mouse retina using TRIzol reagent according to the manufacturer’s protocol. Reverse transcription was performed with a high capacity cDNA reverse transcription kit (Applied Biosystems). Complementary DNA (cDNA) was then used as a template for amplification using the following primers: mouse VEGFR3 (NM_008029.3), forward, 5′-TGTCGAGTGGCTCAAAGGAC-3′ and reverse, 5′-CCAGCTTGCTCACCGTCTTA-3′, and mouse β-Actin (NM_001101), forward, 5′-TGTTTGAGACCTTCAACACC-3′ and reverse, 5′-CGCTCATTGCCGATAGTGAT-3′. The amplification was performed using Gene AMP PCR system 2400 (Applied Biosystems). The amplified PCR products were separated on 1.5% agarose gels, stained with ethidium bromide and the pictures were captured using Amersham Imager 600 GE Healthcare (Piscataway, NJ).

### Western blotting

After appropriate treatments, cell or retinal extracts were prepared and an equal amount of protein from control and each treatment was resolved by electrophoresis on SDS-polyacrylamide gels. The proteins were transferred electrophoretically onto a nitrocellulose membrane. After blocking in 5% (w/v) non-fat dry milk or BSA for 1 h, the membrane was incubated with the indicated primary antibody for overnight at 4 °C. The membrane was then washed with TBST for 3 times, 10 min each, and incubated with horseradish peroxidase-conjugated secondary antibody for 1 h. After washing with TBST for three times, 10 min each, the membrane was incubated with enhanced chemiluminescence detection reagents (Amersham Biosciences) and the antigen-antibody complexes were detected in x-ray film.

### Electrophoretic mobility shift assay (EMSA)

Nuclear extracts of HRMVECs with and without the indicated treatments were prepared using NE-PER nuclear and cytoplasmic extraction kit (Cat. No. 78833, Thermo Scientific) following manufacturer’s instructions. The protein content of nuclear extracts was determined by micro-BCA method (Pierce Biotechnology). Double-stranded oligonucleotides encompassing AP1-binding element at −543 nt (Forward: 5′-GCGATTCTCC**TGCCTCA**GCCCCCCCGAGA-3′ and Reverse: 5′-TCTCGGGGGGGC**TGAGGCA**GGAGAATCGC-3′) were used as a WT probe. Site-directed mutations within AP1-binding element at −543 nt were introduced by Quickchange site-directed mutagenesis kit according to manufacturer’s instructions and using the following primers, Forward: 5′-GCGATTCTCC**TTCGTGA**GCCCCCCCGAGA-3′ and Reverse: 5′-TCTCGGGGGGGC**TCACGAA**GGAGAATCGC-3′ and used as a mutant AP1 probe in EMSA. Both WT and mutant probes were labeled with biotin using 3′-end DNA labeling kit (Cat. No. 89818, Pierce) following the supplier’s instructions. Protein-DNA complexes were formed by incubating 5 µg of nuclear extract in a binding buffer (10 mM Tris-HCl, pH 7.5, 50 mM KCl, 1 mM dithiothreitol, 2 µg of poly(dI-dC) and 2.5% glycerol) with 5 nM of biotin-labeled probe in a total volume of 20 µl for 30 min on ice. The protein-DNA complexes were resolved by electrophoresis on a 6% polyacrylamide gel using Tris-borate-EDTA buffer (44.5 mM Tris-HCl, 44.5 mM borate and 20 mM EDTA, pH 8.0), transferred to Nylon membrane using the same buffer at 100 V for 1 h, UV cross-linked and visualized by chemiluminescence. In the case of supershift EMSA, the complete reaction mixture was incubated with the indicated antibodies for 1 h on ice before separating it by electrophoresis. Normal IgG was used as a negative control.

### Transfections

HRMVECs or MRMVECs were transfected with non-targeted or on-targeted siRNA at final concentration of 100 nM or plasmid DNA at a concentration of 1 μg/ml using Lipofectamine 3000 transfection reagent according to the manufacturer’s instructions. After 6 h of incubation, cells were recovered in EGM for 30 h, synchronized overnight in EBM and used as needed.

### Infections

HRMVECs were infected with Ad-GFP or Ad-dnSTAT3 at final concentration of 40 moi and after 12 h of infection, cells were recovered in EGM for 24 h, synchronized overnight in EBM and used as needed.

### Immunofluorescence staining

At the end of 5 days of hyperoxia period (P7-P12), mice pups were returned to room air. After 72 h of room air (P15), pups were sacrificed, eyes were enucleated, fixed in optimal cutting temperature compound and 8-µm cryosections were made from the central part of the retina. After blocking in normal goat serum, the cryosections were probed with rabbit anti-mouse Ki67 antibody (1:100) or rabbit anti-mouse JunB antibody (1:100), rabbit anti-mouse VEGFR2 antibody (1:100) or rabbit anti-mouse VEGFR3 antibody (1:100) in combination with rat anti-mouse CD31 antibody (1:100) followed by incubation with Alexa Fluor 568-conjugated goat anti-rabbit and Alexa Fluor 488-conjugated goat anti-rat secondary antibodies. The sections were observed under Zeiss inverted microscope (Axiovision Observer.z1; 40×/NA 0.6 or 10×/NA 0.45) and the fluorescence images were captured by Zeiss AxioCam MRm camera using the microscope operating and image analysis software AxioVision 4.7.2 (Carl Zeiss Imaging Solutions GmbH).

### Statistics

All experiments were repeated 3 times and the data are presented as mean ± SD. Normality of the data (using D’Agostino-Pearson normality test) and the equality of group variance (using F test) were performed on all data using GraphPad Prism v 8.00 software. The normally distributed data with similar variance were analyzed by One-way ANOVA followed by Fisher’s least significant difference post hoc test or two-tailed student “*t*” test and the *p* values <0.05 considered statistically significant. Samples sizes were estimated based on previous experiments^18,20^.

## Results

### PKCθ mediates VEGFA-induced HRMVEC migration, sprouting and tube formation

**Fig. 1:**
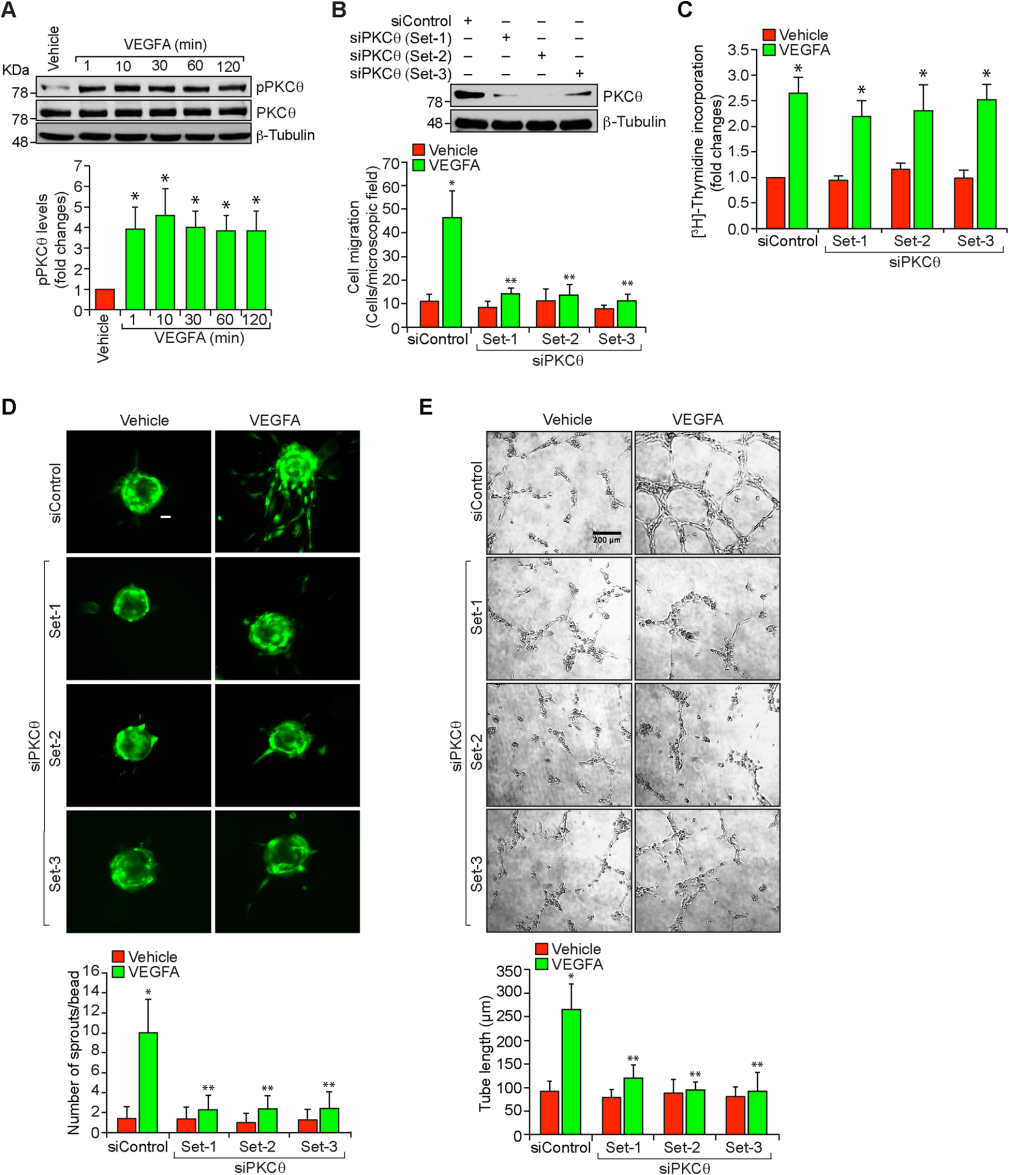
PKCθ mediates VEGFA-induced angiogenic events in HRMVECs. **a** Western blot analysis of control and various time periods of VEGFA (40 ng/ml)-treated HRMVECs for phosphorylated PKCθ levels. The blot was normalized to total PKCθ levels and β-tubulin. **b** Upper panel: Western blot analysis of PKCθ and β-tubulin levels to show the specificity and efficacy of siControl and siPKCθ (100 nM) in HRMVECs. Bottom panel: The effect of siControl and siPKCθ on VEGFA (40 ng/ml)-induced HRMVEC migration using Boyden chamber assay. **c**–**e** All the conditions were same as in (**b**) except that cells were treated with and without VEGFA (40 ng/ml) and DNA synthesis (**c**), sprouting (**d**) or tube formation (**e**) were measured. The bar graphs represent quantitative analysis of three independent experiments. The values are presented as mean ± SD. *p < 0.01 vs vehicle control or siControl; **p < 0.01 vs siControl + VEGFA. Scale bars in (**d**) and (**e**) are 50 and 200 μm, respectively.

VEGFA plays a major role in both developmental and pathological angiogenesis by stimulating endothelial cell functions required for new blood vessel formation, such as migration, proliferation, differentiation and survival^23^. Retinal neovascularization is a clinical manifestation of diabetic retinopathy, and it was reported that genetic deletion of PKCθ protects mice against diet-induced insulin resistance^16^. Therefore, we asked the question whether PKCθ plays a role in retinal neovascularization. VEGFA stimulated PKCθ phosphorylation in a time dependent manner with maximum effect at 10 min and sustaining thereafter in HRMVECs (Fig. 1a). In addition, siRNA-mediated downregulation of PKCθ levels inhibited VEGFA-induced HRMVEC migration, sprouting, and tube formation with little or no effect on DNA synthesis (Fig. 1b–e). These results suggest that PKCθ activation is necessary for VEGFA-induced HRMVEC migration, sprouting, and tube formation but not proliferation.

### Hypoxia induced retinal neovascularization requires PKCθ activation

**Fig. 2:**
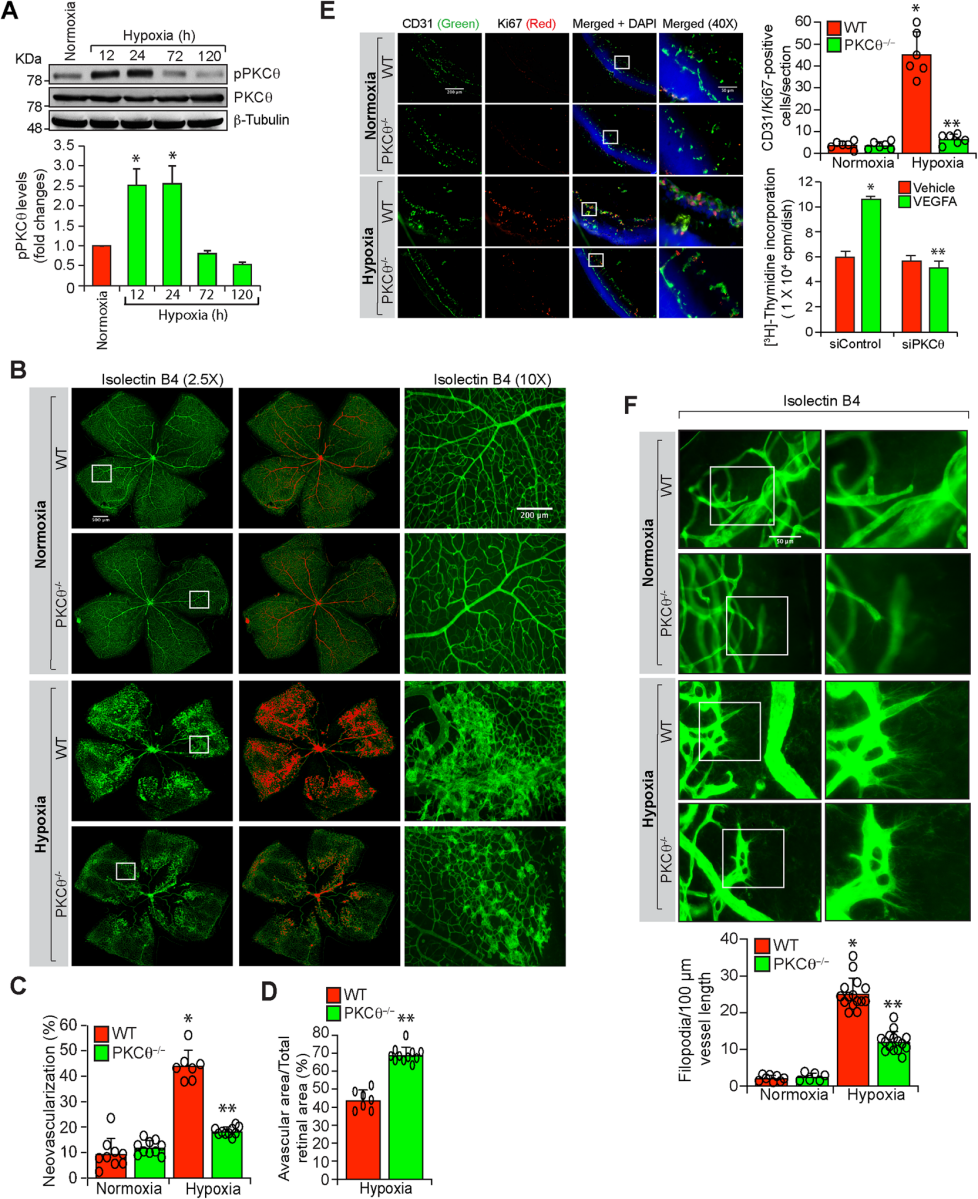
PKCθ mediates hypoxia-induced retinal neovascularization. **a** Western blot analysis of retinal extracts of control and the indicated time periods of hypoxic C57BL/6 (WT) mice pups for phosphorylated PKCθ levels. The blot was normalized to total PKCθ levels and β-tubulin. **b** Isolectin B4 staining of retinal flat mounts of normoxic and 5 days of hypoxic WT and PKCθ^−/−^ mice pups. Retinal vascularization is shown in the first column at 2.5× magnification (scale bar, 500 μm). Neovascularization is highlighted in red in the second column. The third column shows the selected rectangular areas of the images in the first column at 10× magnification (scale bar, 200 μm). **c**,**d** Retinal neovascularization (**c**) and avascular area (**d**) were determined as described in “Materials and Methods.” **e** Left panel: Double immunofluorescence staining of retinal cross sections of normoxic and 3 days of hypoxic WT and PKCθ^−/−^ mice pups for CD31 and Ki67. The extreme right column shows 40× magnification of the areas selected by rectangular boxes in the immediate left column images (scale bars in the far left and far right columns are 200 and 50 μm, respectively). Retinal EC proliferation was measured by counting CD31 and Ki67-positive cells that extended anterior to the inner limiting membrane in each section (*n* = 6 eyes, 3 sections/eye). Upper right panel: Quantitative analysis of CD31 and Ki67-positive cells. Bottom right panel: The effect of siControl and siPKCθ on VEGFA (40 ng/ml)-induced DNA synthesis in MRMVECs. **f** Upper panel: All the conditions were the same as in (**b**) except that the retinal flat mounts were examined for EC filopodia at 40× magnification (scale bar, 50 μm). Bottom panel: Quantitative analysis of number of filopodia/unit vessel length. The bar graphs represent quantitative analysis of three blots or 6 retinas. The values are presented as mean ± SD. *p < 0.01 vs normoxia or siControl; **p < 0.01 vs WT hypoxia or siControl + VEGFA.

Based on in vitro findings, we next studied the role of PKCθ in retinal neovascularization using a mouse model of oxygen-induced retinopathy (OIR). Retinal extracts of normoxic and various time periods of hypoxic C57BL/6 (WT) mice pups were analyzed by western blotting for PKCθ phosphorylation. As compared to normoxic control, hypoxia induced PKCθ phosphorylation at 12 and 24 h in the retina (Fig. 2a). Furthermore, genetic deletion of PKCθ substantially reduced hypoxia-induced retinal neovascularization with reduction in tufts and anastomoses and increased avascular area as compared to WT mice pups (Fig. 2b–d). In addition, PKCθ deletion inhibited hypoxia-induced retinal EC proliferation as observed by a decrease in the number of Ki67 and CD31-positive cells, markers for proliferation and ECs, respectively (Fig. 2e). Depletion of PKCθ levels using its siRNA also reduced VEGFA-induced DNA synthesis in mouse retinal microvascular endothelial cells (MRMVECs) (Fig. 2e). Furthermore, hypoxic retina of PKCθ^−/−^ mice pups showed decreased EC filopodia formation as compared to hypoxic retina of WT mice pups, suggesting a possible role of PKCθ in tip cell formation (Fig. 2f). These findings indicate that activation of PKCθ is required for hypoxia-induced retinal EC proliferation, tip cell formation and neovascularization.

### PKCθ-mediated JunB expression is required for VEGFA-induced HRMVEC migration, sprouting and tube formation

**Fig. 3:**
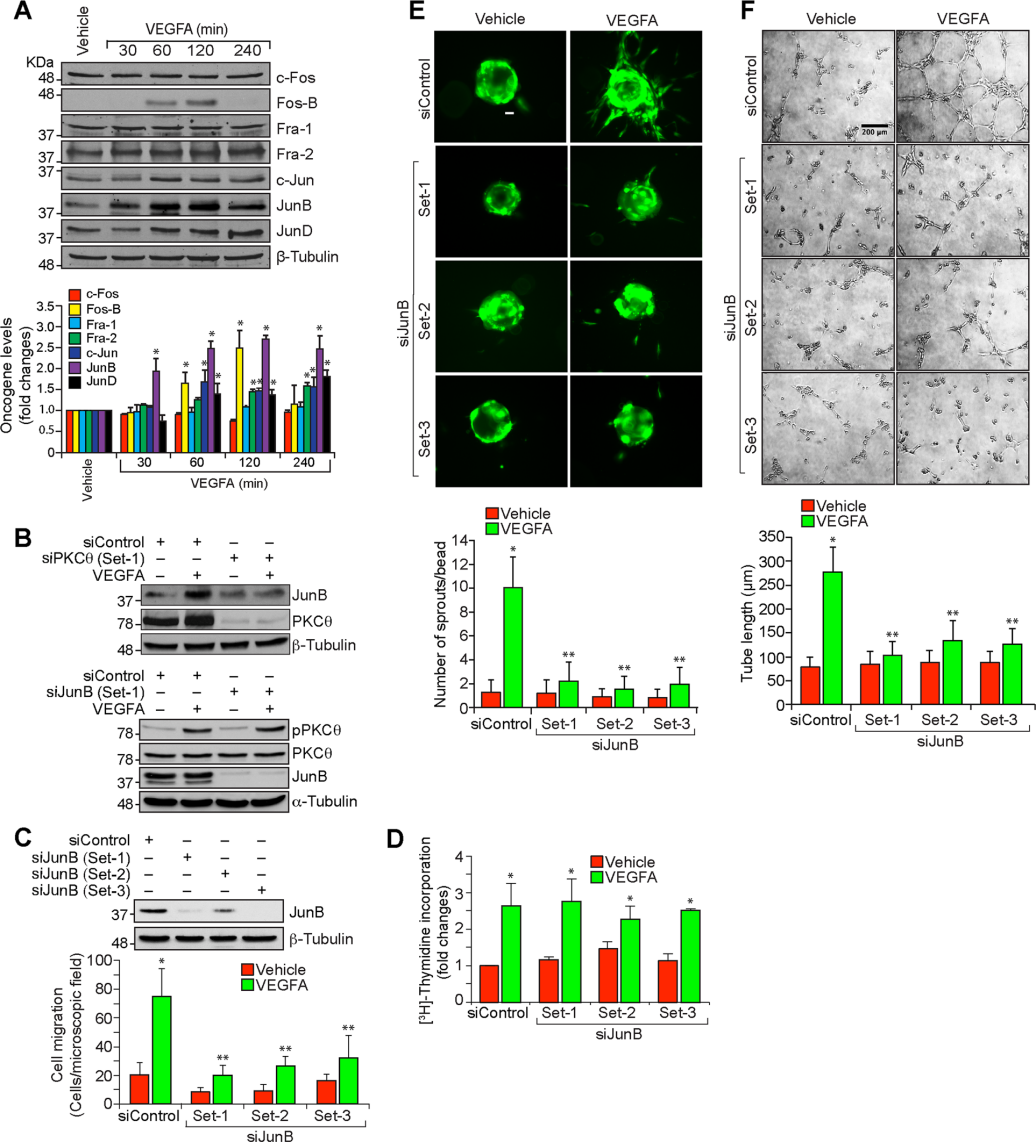
JunB mediates VEGFA-induced angiogenic events in HRMVECs. **a** Western blot analysis of control and various time periods of VEGFA (40 ng/ml)-treated HRMVECs for the indicated proteins. **b** The effect of siControl, siPKCθ and siJunB (100 nM) on VEGFA (40 ng/ml)-induced JunB expression at 2 h and PKCθ phosphorylation at 10 min. The blot was sequentially reprobed for PKCθ, JunB, β-tubulin and α-tubulin levels to show the specificity and efficacy of the siRNA on its target and off target molecules. **c** Upper panel: Western blot analysis of JunB and β-tubulin levels to show the specificity and efficacy of siControl and siJunB (100 nM) in HRMVECs. Bottom panel: The effect of siControl and siJunB on VEGFA (40 ng/ml)-induced HRMVEC migration. **d**–**f** All the conditions were same as in (**c**) except that cells were treated with and without VEGFA (40 ng/ml) and DNA synthesis (**d**), sprouting (**e**) or tube formation (**f**) were measured. The bar graphs represent quantitative analysis of three independent experiments. The values are presented as mean ± SD. *p < 0.01 vs vehicle control or siControl; **p < 0.01 vs siControl + VEGFA. Scale bars in (**e**) and (**f**) are 50 and 200 μm, respectively.

PKC isoforms have been reported to mediate activation of various transcription factors including AP1^24,25^. In addition, we have previously shown that induction of c-Jun and Fra1 is required for adaptative angiogenesis in a hindlimb ischemia model^18^. Based on these clues, we wanted to test whether PKCθ influences AP1 in the modulation of retinal neovascularization. To address this point, we tested the effect of VEGFA on the expression of the Fos and Jun family of the transcriptional factor, AP1. VEGFA induced JunB expression very robustly as compared to all the other Jun and Fos family of the protooncogenes in HRMVECs (Fig. 3a). Furthermore, depletion of PKCθ levels by its siRNA inhibited VEGFA-induced JunB expression in HRMVECs (Fig. 3b). On the other hand, depletion of JunB levels had no effect on VEGFA-induced PKCθ phosphorylation, suggesting that JunB is a downstream target of PKCθ (Fig. 3b). In regard to its function, downregulation of JunB levels by its siRNA attenuated VEGFA-induced HRMVEC migration, sprouting and tube formation while having no effect on DNA synthesis (Fig. 3c–f). Together, these observations reveal that PKCθ-mediated JunB expression is required for VEGFA-induced angiogenic events of HRMVECs.

### PKCθ-mediated JunB expression is required for retinal neovascularization

**Fig. 4:**
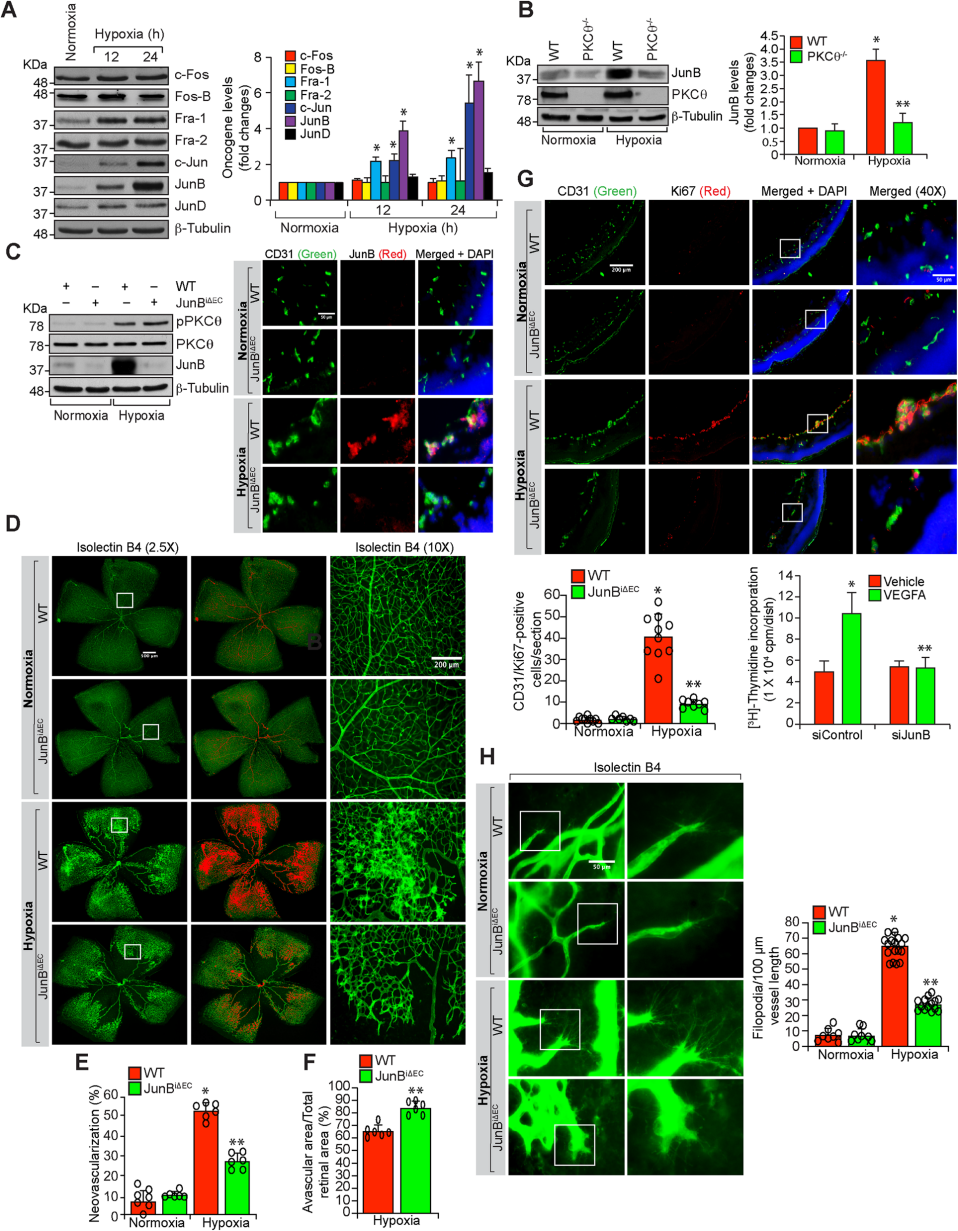
Endothelial-specific deletion of JunB negates hypoxia-induced retinal neovascularization. **a** Western blot analysis of retinal extracts of normoxic and 12 h and 24 h of hypoxic WT mice pups for the indicated proteins. **b** Western blot analysis of retinal extracts of normoxic and 24 h of hypoxic WT and PKCθ^−/−^ mice pups for JunB levels. The blot was subsequently reprobed for PKCθ and β-tubulin levels. **c** Left panel: Normoxic or hyperoxic JunB^flox/flox^:Cdh5-Cre^ERT2^ mice pups at P10 and P11 were injected with 100 μg tamoxifen intraperitoneally, and normoxic and 24 h of hypoxic (P13) pup retinal extracts were prepared and analyzed by western blotting for phospho and total PKCθ, JunB and β-tubulin levels. Right panel: All the conditions were the same as in the left panel except that eyes from normoxic and 72 h of hypoxic pups were enucleated, fixed, sections were made and co-immunostained for CD31 and JunB. **d** All the conditions are same as in right (**c**) except that eyes were enucleated from normoxic and 120 h of hypoxic pups, fixed, retinas isolated, stained with isolectin B4, flat mounts were made and retinal vascularization and neovascularization were measured. Retinal vascularization is sown in the first column at 2.5× magnification (scale bar 500 μm) and neovascularization is highlighted in red in the second column. The third column shows the selected rectangular areas of the images in the first column at 10× magnification (scale bar, 200 μm). **e**,**f** Retinal neovascularization (**e**) and avascular area (**f**) were determined as described in “Materials and Methods”. **g** Upper panel: All the conditions were the same as in left (**c**) except that sections were co-immunostained for CD31 and Ki67. The extreme right column shows 40× magnification of the areas selected by rectangular boxes in the immediate left column images (scale bars in the far left and far right columns are 200 and 50 μm, respectively). Retinal EC proliferation was measured by counting CD31 and Ki67-positive cells from the inner limiting membrane to the extended region in each section (*n* = 6 eyes, 3 sections/eye). Bottom left panel: Quantitative analysis of CD31 and Ki67-positive cells. Bottom right panel: The effect of siControl and siJunB on VEGFA (40 ng/ml)-induced DNA synthesis in MRMVECs. **h** All the conditions were the same as in (**d**) except that the retinal flat mounts were examined for filopodia at 40× magnification (scale bar, 50 μm). The bar graphs represent quantitative analysis of three blots or 7 retinas. The values are presented as mean ± SD. *p < 0.01 vs normoxia or WT normoxia or siControl; **p < 0.01 vs WT hypoxia, siControl + hypoxia or siControl + VEGFA.

Based on the role of JunB in HRMVEC migration, sprouting and tube formation, we next examined its role in retinal neovascularization. Hypoxia induced JunB expression more robustly than c-Jun or JunD or the Fos family of the AP1 transcriptional factors in mouse retina (Fig. 4a). To understand the mechanisms by which JunB was induced in the hypoxic retina, we next tested the role of PKCθ. As expected, hypoxia induced JunB expression as compared to normoxia in the retina of WT mice pups (Fig. 4b). However, hypoxia had no effect on JunB expression in the retina of PKCθ^−/−^ mice pups (Fig. 4b). In addition, endothelial-specific deletion of JunB had no effect on hypoxia-induced PKCθ phophorylation (Fig. 4c). Furthermore, as revealed by coimmunostaining, hypoxia-induced JunB expression occurred mostly in ECs (Fig. 4c). These findings imply that hypoxia-induced JunB expression occurs in retinal ECs and was dependent on activation of PKCθ. Consistent with these obervations, endothelial-specific deletion of JunB substantially reduced retinal neovascularization with decreased endothelial cell proliferation, tufts and filopodia and increased avascular area compared to WT mice pups (Fig. 4d–h). Depletion of JunB by its siRNA also inhibited VEGFA-induced DNA synthesis in MRMVECs (Fig. 4g, bottom right panel). These results indicate that hypoxia-induced JunB expression in ECs was dependent on PKCθ activation and is required for retinal neovascularization.

### PKCθ-JunB axis enhances VEGFR3 expression in the mediation of retinal neovascularization

**Fig. 5:**
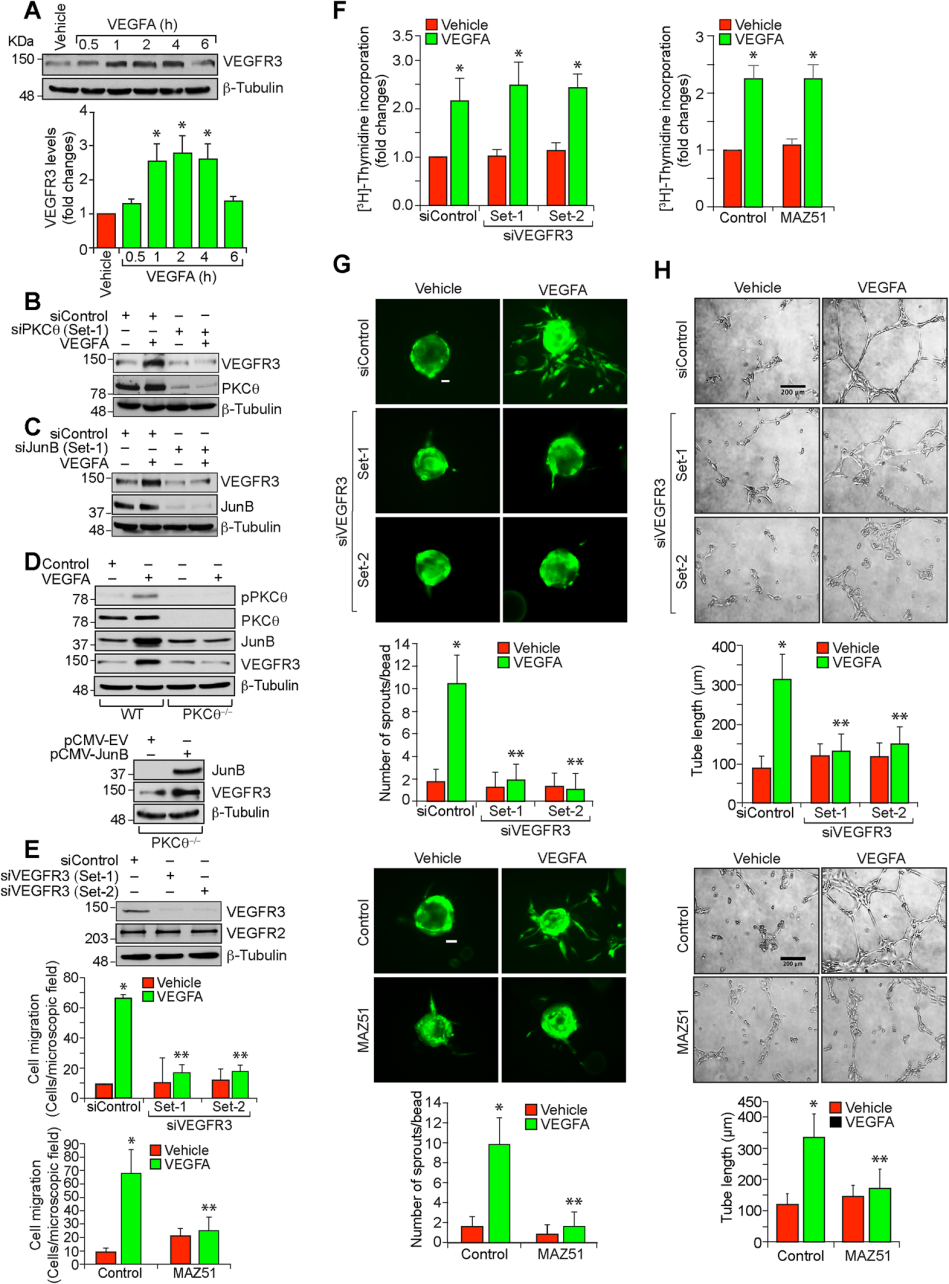
VEGFR3 mediates VEGFA-induced angiogenic events in HRMVECs. **a** Western blot analysis of control and the indicated time periods of VEGFA (40 ng/ml)-treated HRMVECs for VEGFR3 and β-tubulin levels. **b**,**c** The effect of siControl, siPKCθ and siJunB (100 nM) on VEGFA (40 ng/ml)-induced (2 h) VEGFR3 levels. The blots were sequentially reprobed for PKCθ and β-tubulin levels or JunB and β-tubulin levels to show the specificity and efficacy of siRNA on its target and off target molecules. **d** Upper panel: Retinal ECs were isolated from WT and PKCθ^−/−^ mice and tested for the effect of VEGFA on PKCθ phosphorylation and JunB and VEGFR3 expression. Lower panel: Retinal ECs from PKCθ^−/−^ mice were transfected with empty vector or JunB expression vector and two days later cell extracts were prepared and analyzed by western blotting for JunB, VEGFR3 and β-tubulin levels. **e** Upper panel: western blot analysis of VEGFR2, VEGFR3 and β-tubulin levels to show the specificity and efficacy of siControl and siVEGFR3 (100 nM) in HRMVECs. Bottom panel: The effect of siControl, siVEGFR3 and MAZ51 (5 μM) on VEGFA (40 ng/ml)-induced HRMVEC migration. **f**–**h** All the conditions were same as in (**e**) except that cells were treated with and without VEGFA (40 ng/ml) and DNA synthesis (**f**), sprouting (**g**) or tube formation (**h**) were measured. The bar graphs represent quantitative analysis of three independent experiments. The values are presented as mean ± SD. *p < 0.01 vs vehicle control or siControl; **p < 0.01 vs siControl + VEGFA. Scale bars in (**g**) and (**h**) are 50 and 200 μm, respectively.

**Fig. 6:**
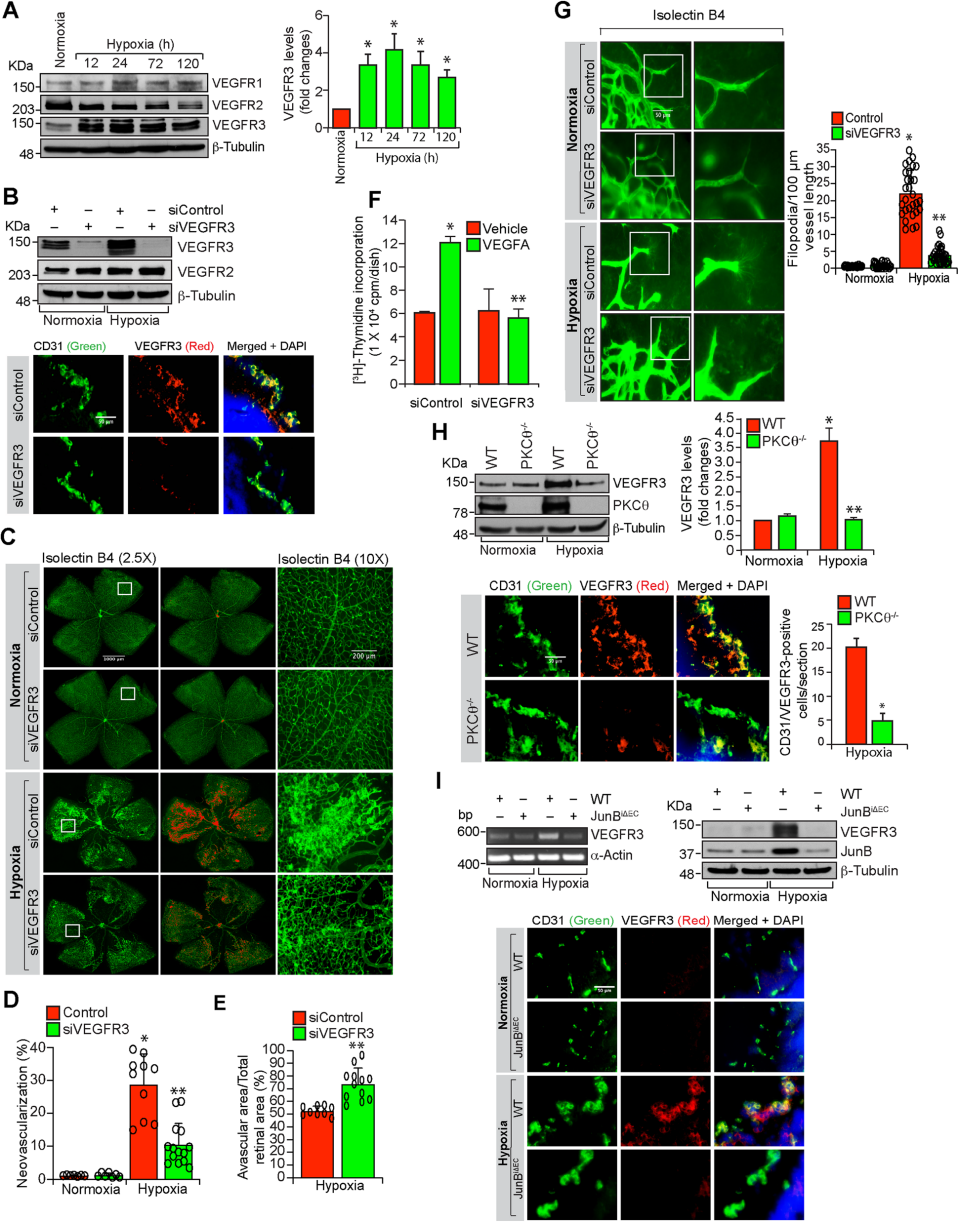
VEGFR3 mediates hypoxia-induced retinal neovascularization. **a** Western blot analysis of retinal extracts of normoxic and the indicated time periods of hypoxic WT mice pups for VEGFR1, VEGFR2, VEGFR3 and β-tubulin levels. **b** Upper panel: Mice pups were injected intravitreally with 1 μg/0.5 μl/eye of siControl or siVEGFR3 at P11 and P12 and at P13, retinal extracts were prepared and analyzed by western blotting for VEGFR2, VEGFR3 and β-tubulin levels. Bottom panel: All the conditions were the same as in upper panel except that pups were received siRNA at P11, P12 and P13 and at P15, eyes were enucleated, fixed, sections were made and immunostained for CD31 and VEGFR3. **c** All the conditions are same as in bottom (**b**) except that eyes were enucleated at P17, fixed, retinas were isolated, stained with isolectin B4 and flat mounts were made. Retinal vascularization is shown in the first column at 2.5× magnification (scale bar, 1000 μm). Neovascularization is highlighted in red in the second column. The third column shows the selected rectangular areas of the images in the first column at 10× magnification (scale bar, 200 μm). **d**,**e**. Retinal neovascularization (**d**) and avascular area (**e**) were determined as described in “Materials and Methods.” **f** The effect of siControl and siVEGFR3 on VEGFA (40 ng/ml)-induced DNA synthesis in MRMVECs. **g** Left panel: All the conditions were the same as in (**c**) except that the retinal flat mounts were examined for filopodia at 40× magnification (scale bar, 50 μm). Right panel: Quantitative analysis of number of filopodia/unit vessel length. **h** Upper left panel: western blot analysis of normoxic and 24 h of hypoxic retina of WT and PKCθ^−/−^ mice pups for VEGFR3, PKCθ and β-tubulin levels. Bottom left panel: All the conditions were the same as in upper panel except that eyes were enucleated at 3 days of hypoxia (P15), fixed, sections were made and immunostained for CD31 and VEGFR3. Upper and bottom right panels: Quantitative analysis of VEGFR3 levels and CD31 and VEGFR3-positive cells, respectively. **i** Upper left panel: Normoxic or hyperoxic JunB^flox/flox^:Cdh5-Cre^ERT2^ mice pups at P10 and P11 were injected with 100 μg tamoxifen intraperitoneally, and RNA was isolated from normoxic and 24 h of hypoxic (P13) pup retinas and analyzed by RT-PCR for VEGFR3 and α-actin mRNA levels. Upper right panel: All the conditions were the same as in the left panel except that retinal extracts were prepared and analyzed for western blotting for VEGFR3, JunB and β-tubulin levels. Lower panel: All the conditions were the same as in the upper panels except that eyes were enucleated, fixed, sections were made and co-immunostained for CD31 and VEGFR3. The bar graphs represent quantitative analysis of three blots or 7 retinas. The values are presented as mean ± SD. *p < 0.01 vs normoxia or WT + normoxia or siControl + normoxia; **p < 0.01 vs WT + hypoxia or siControl + hypoxia.

In order to identify the target genes of JunB, we studied the effect of VEGFA on the expression VEGFR3. VEGFA induced VEGFR3 expression in a time dependent manner and this effect was negated by depletion of PKCθ or JunB levels (Fig. 5a–c). To substantiate the role of endothelial-specific PKCθ-JunB axis in VEGFR3 expression, retinal ECs from WT and PKCθ^−/−^ mice were isolated and tested for the effect of VEGFA. First, as expected, VEGFA stimulated PKCθ phosphorylation only in retinal ECs of WT but not PKCθ^−/−^ mice (Fig. 5d); second, VEGFA induced JunB and VEGFR3 expression again only in retinal ECs of WT but not PKCθ^−/−^ mice (Fig. 5d); and third, overexpression of JunB restored VEGFR3 expression in retinal ECs of PKCθ^−/−^ mice (Fig. 5d). Furthermore, VEGFA induced migration, sprouting and tube formation of retinal ECs isolated from WT mice but not PKCθ^−/−^ mice (Supplementary Fig. 1A–C). In addition, overexpression of JunB restored migration, sprouting and tube formation in retinal ECs of PKCθ^−/−^ mice (Supplementary Fig. 1A–C). Based on these findings, we next studied the role of VEGFR3 in angiogenic responses of HRMVECs. Interestingly, either siRNA-mediated depletion of VEGFR3 levels or pharmacological inhibition of its function by MAZ51^26^ while having no effect on DNA synthesis attenuated VEGFA-induced HRMVEC migration, sprouting, and tube formation (Fig. 5e–h). To understand the role of VEGFR3 in hypoxia-induced retinal neovascularization, we first studied the effect of hypoxia on VEGFR1, VEGFR2 and VEGFR3 expression. Hypoxia while having no major effect on VEGFR1 levels induced VEGFR3 expression more robustly and decreased VEGFR2 levels in a time dependent manner in the mouse retina (Fig. 6a). In addition, depletion of VEGFR3 levels by its siRNA reduced retinal neovascularization with decreased endothelial cell proliferation, decreased number of tufts and filopodia and increased avascular area as compared to mice pups injected with control siRNA (Fig. 6b–g). Furthermore, genetic deletion of PKCθ significantly reduced hypoxia-induced VEGFR3 expression in the retina, mostly in ECs (Fig. 6h). Similarly, endothelial-specific deletion of JunB also suppressed hypoxia-induced VEGFR3 expression both at mRNA and protein levels in retinal endothelial cells (Fig. 6i). These results indicate that PKCθ-JunB axis mediates hypoxia-induced VEGFR3 expression in ECs promoting retinal neovascularization.

### JunB binds to VEGFR3 promoter in response to VEGFA

To understand the molecular mechanisms by which JunB regulates VEGFA-induced VEGFR3 expression, we identified one potential AP1-binding site at −543 nt in VEGFR3 promoter by TRANSFAC analysis (Supplementary Fig. 2A). We next studied the time course effect of VEGFA on AP1-DNA binding activity by EMSA using AP1-binding element at −543 nt as a biotin-labeled double-stranded oligonucleotide probe. VEGFA induced AP1-DNA-binding activity in a time dependent manner in HRMVECs (Supplementary Fig. 2B). Furthermore, no AP1-DNA binding activity was observed in the nuclear extracts of VEGFA-treated HRMVECs when AP1 element at -543 nt was mutated (Supplementary Fig. 2C). In addition, supershift EMSA using anti-JunB, anti-c-Jun or anti-JunD antibodies showed the presence of only JunB but not c-Jun or junD in VEGFA-induced AP1-DNA complexes (Supplementary Fig. 2D). Besides, depletion of PKCθ levels using its siRNA reduced VEGFA-induced AP1-DNA binding activity (Supplementary Fig. 2E). These findings infer that JunB mediates VEGFA-induced AP1-VEGFR3 promoter binding activity and this effect was dependent on activation of PKCθ.

### VEGFR2 is essential for VEGFA and hypoxia-induced VEGFR3 expression

**Fig. 7:**
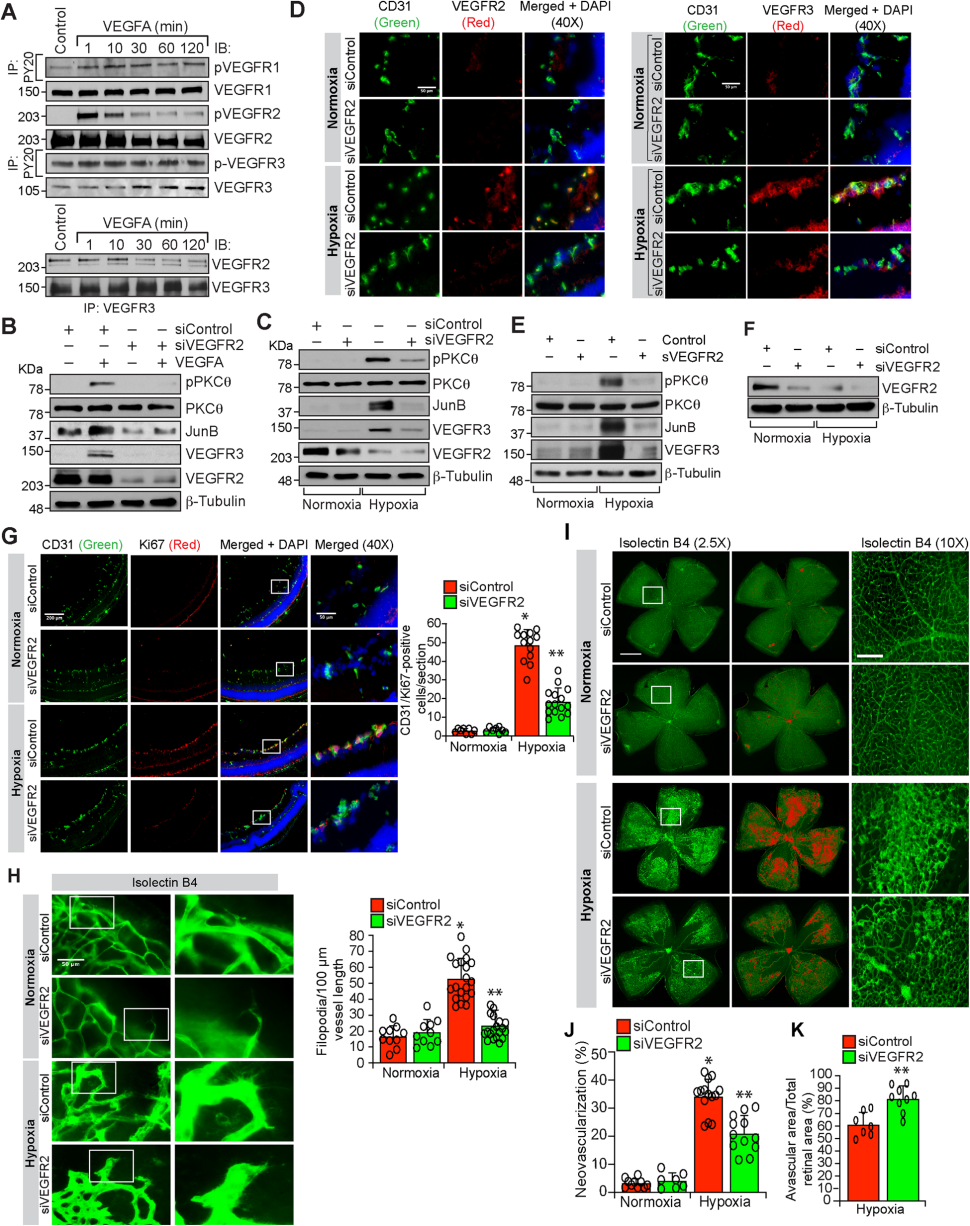
Depletion of VEGFR2 levels blunts retinal neovascularization. **a** Upper panel: Equal amount of protein from control and the indicated time periods of VEGFA-treated HRMVECs were analyzed for phospho and total VEGFR1, 2 and 3 levels. Lower panel: The cell extracts were analyzed for VEGFR2 and VEGFR3 complex formation. **b** HRMVECs were transfected with siControl or siVEGFR2 (100 nM), quiesced, treated with and without VEGFA (40 ng/ml) for 10 min (for pPKCθ) or 120 min (for JunB and VEGFR3) and cell extracts were prepared and analyzed by western blotting for the indicated proteins. **c** Retinas from normoxic and 24 h (i.e., at P13) of hypoxic WT mice pups that received siControl or siVEGFR2 (1 μg/0.5 μl/eye) by intravitreal injections at P10 and P11 were isolated, extracts were prepared and analyzed by western blotting for the indicated proteins. **d** Eyes from normoxic and 72 h (i.e., at P15) of hypoxic WT mice pups that received siControl or siVEGFR2 (1 μg/0.5 μl/eye) by intravitreal injections at P11, P12 and P13 were enucleated, fixed, sections were made and coimmunostained for CD31 and VEGFR2 (left panel) and CD31 and VEGFR3 (right panel). **e** Eyes from normoxic and 24 h (i.e., at P13) of hypoxic WT mice pups that were injected intravitreally with vehicle or 0.05 μg/0.5 μl/eye of soluble VEGFR2 at P11 and P12 were enucleated, retinas were isolated, protein extracts were prepared and analyzed by western blotting for the indicated proteins using their specific antibodies. **f** Eyes from normoxic and 24 h of hypoxic mice pups that were injected intravitreally with siControl or siVEGFR2 (0.5 μg/0.5 μl/eye) at P11 and P12 were enucleated, retinas were isolated, protein extracts were prepared and analyzed by western blotting for VEGFR3 levels using its specific antibodies and the blot was normalized for β-tubulin. **g** All the conditions were the same as in (**d**) except that sections were coimmunostained for CD31 and Ki67. The bar graph shows quantification of proliferating ECs per section. **h** All the conditions were the same as in (**d**) except that eyes were enucleated at P17, fixed, retinas were isolated, stained with isolectin B4, flat mounts were made and examined for filopodia at 40× magnification (scale bar, 50 μm). Bar graph shows quantification of the number of filopodia/unit vessel length. **i** All the conditions were the same as in (**h**) except that the flat mounts were examined for retinal vascularization. Retinal vascularization is shown in the first column at 2.5× magnification (scale bar, 500 μm). Neovascularization is highlighted in red in the second column. The third column shows the selected rectangular areas of the images in the first column at 10× magnification (scale bar, 200 μm). **j**,**k** Retinal neovascularization (**j**) and avascular area (**k**) were determined as described in “Materials and Methods.” The values are presented as mean ± SD. *p < 0.01 vs normoxia; **p < 0.01 vs siControl + hypoxia.

**Fig. 8:**
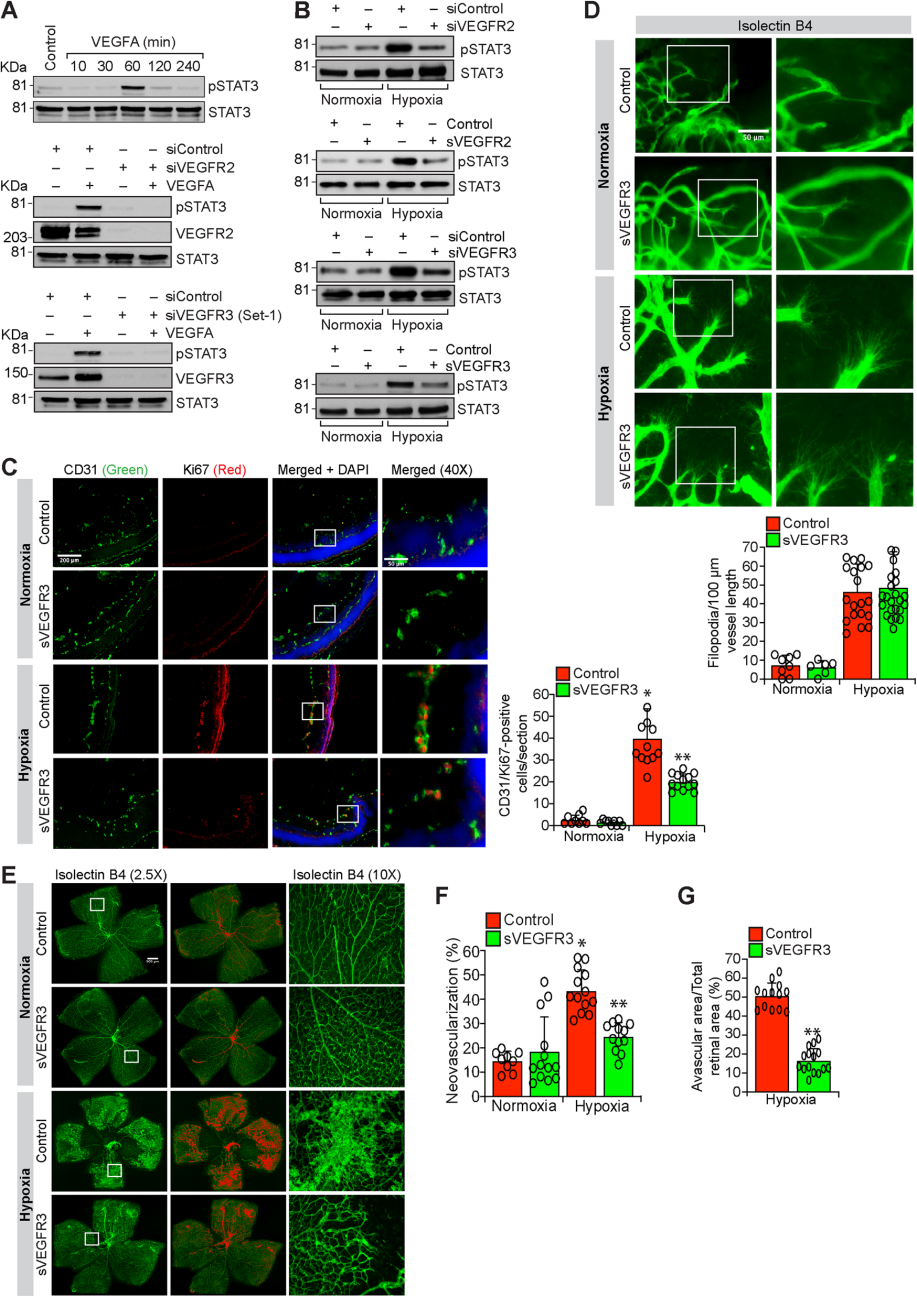
VEGFR3 mediates retinal neovascularization in ligand dependent and independent manner. **a** Upper panel: western blot analysis of control and the indicated time periods of VEGFA (40 ng/ml)-treated HRMVECs for phospho (Y705) and total STAT3 levels. Middle and bottom panels: The effect of siControl, siVEGFR2 and siVEGFR3 (100 nM) on VEGFA (40 ng/ml)-induced STAT3 phosphorylation. The blots were sequentially reprobed for total STAT3, VEGFR2 or VEGFR3 levels to show the loading control and the specificity and efficacy of siRNAs on their target molecules. **b** Upper two panels: Eyes from normoxic and 24 h (i.e., at P13) of hypoxic WT mice pups that were injected intravitreally with siControl, siVEGFR2 (0.5 μg/0.5 μl/eye) or sVEGFR2 (0.05 μg/0.5 μl/eye) at P11 and P12 were enucleated, retinas were isolated, protein extracts were prepared and analyzed by western blotting for phospho and total STAT3 levels using their specific antibodies. Lower two panels: Eyes from normoxic and 24 h (i.e., at P13) of hypoxic WT mice pups that were injected intravitreally with siControl, siVEGFR3 (0.5 μg/0.5 μl/eye) or sVEGFR3 (0.05 μg/0.5 μl/eye) at P11 and P12 were enucleated, retinas were isolated, protein extracts were prepared and analyzed by western blotting for phospho and total STAT3 levels using their specific antibodies. **c** Mice pups were injected intravitreally with vehicle or 0.05 μg/0.5 μl/eye of soluble VEGFR3 at P11, P12 and P13 and normoxic and 72 h of hypoxic eyes were enucleated, fixed, sections were made and coimmunostained for CD31 and Ki67. **d** All the conditions were the same as in (**c**) except that eyes were enucleated at 120 h of hypoxia (i.e., at P17), fixed, retinas were isolated, stained with isolectin B4, flat mounts were made and examined for filopodia at 40× magnification (scale bar, 50 μm). Bar graph shows quantitative analysis of the number of filopodia/unit vessel length. **e** All the conditions were the same as in (**d**) except that the flat mounts were examined for retinal vascularization. Retinal vascularization is shown in the first column at 2.5× magnification (scale bar, 500 μm). Neovascularization is highlighted in red in the second column. The third column shows the selected rectangular areas of the images in the first column at 10× magnification (scale bar, 200 μm). **f**,**g** Retinal neovascularization (**f**) and avascular area (**g**) were determined as described in “Materials and Methods.” The values are presented as mean±SD. *p < 0.01 vs normoxia; **p < 0.01 vs control + hypoxia.

To understand the upstream mechanisms by which VEGFA or hypoxia regulates VEGFR3 expression, we studied the role of VEGFR2. First, VEGFA while having no significant effect on VEGFR3 phosphorylation induced VEGFR1 and VEGFR2 phosphorylation in a time dependent manner (Fig. 7a). Furthermore, VEGFR2 and VEGFR3 were found to exist in complex under basal condition and in response to VEGFA they were dissociated from each other (Fig. 7a). Second, depletion of VEGFR2 levels by its siRNA completely reduced VEGFA-induced PKCθ activation, and JunB and VEGFR3 expression in HRMVECs (Fig. 7b). Similarly, VEGFR2 knockdown reduced hypoxia-induced PKCθ activation and JunB and VEGFR3 expression in the retina of WT mice pups (Fig. 7c). Coimmunostaining for CD31 and VEGFR3 shows that hypoxia induces VEGFR3 expression predominantly in retinal ECs and depletion of VEGFR2 levels by its siRNA suppresses this effect (Fig. 7d). Furthermore, soluble VEGFR2 also attenuated hypoxia-induced VEGFR3 expression in the retina of WT mice pups (Fig. 7e). In addition, downregulation of VEGFR2 levels by its siRNA or blockade of VEGFR2 signaling by soluble VEGFR2 inhibited hypoxia-induced retinal EC proliferation, filopodia formation and neovascularization (Fig. 7f–k and Supplementary Fig. 3A–E). These results clearly demonstrate that VEGFA or hypoxia-induced VEGFR3 expression requires activation of VEGFR2 upstream to PKCθ-JunB signaling both in HRMVECs and mouse retina mediating retinal neovascularization. The next obvious question we asked is how VEGFR3 is involved in VEGFA/VEGFR2-induced retinal neovascularization. Towards this end, we looked for potential downstream target signaling molecules of VEGFR3 and found that VEGFA stimulates tyrosine phosphorylation of STAT3 in HRMVECs in a delayed manner and this response requires the involvement of both VEGFR2 and VEGFR3 as depletion of either receptor level by their respective siRNA inhibited VEGFA-induced STAT3 phosphorylation (Fig. 8a). Similar to these observations, either depletion of VEGFR2 or blockade of its function by its soluble receptor protein substantially attenuated hypoxia-induced STAT3 phosphorylation (Fig. 8b, upper two panels). Depletion of VEGFR3 levels by its siRNA or inhibition of its function by its soluble receptor protein also attenuated hypoxia-induced STAT3 phosphorylation (Fig. 8b, lower two panels). In regard to the role of STAT3 in retinal angiogenesis, blockade of STAT3 signaling by expression of its dominant negative mutant^27^ suppressed VEGFA-induced HRMVEC migration, sprouting and tube formation in vitro and hypoxia-induced retinal EC proliferation, filopodia formation and neovascularization in vivo (Supplementary Figs 4A–D and 5A–E). To test whether VEGFR3 requires ligand binding in the modulation of retinal neovascularization, we studied the effects of soluble VEGFR3 protein. Administration of soluble VEGFR3 protein while having little effect on filopodia formation substantially attenuated hypoxia-induced retinal EC proliferation and neovascularization (Fig. 8c–g). These observations indicate that VEGFR3 acts via ligand dependent and independent manner in mediating VEGFA-VEGFR2 effects on retinal neovascularization.

## Discussion

VEGF is a major growth factor that regulates angiogenesis during development and disease^23^. Various clinical studies have shown an elevated intraocular VEGFA levels in patients with retinopathy of prematurity, diabetes mellitus, retinal vein occlusion, and macular degeneration^28,29^. Besides, the FDA-approved treatments for retinal neovascularization are based on the inhibition of VEGF function^28^. It was well established that VEGFR2 mediates most of the cellular effects of VEGFA during developmental and pathological angiogenesis^6,30,31^. In addition, many studies have shown that PKC isoforms such as PKCα, PKCβ and PKCζ modulate VEGFA-induced EC proliferation^15,32^. In the present study, we show that VEGFA activates PKCθ in mediating HRMVEC migration, sprouting and tube formation, suggesting the role of PKCθ in VEGFA-induced angiogenesis. Similar to our findings, other studies have also demonstrated that PKCθ might be involved in endothelial cell proliferation and migration perhaps via its role in actin polymerization^33,34^. Retinal neovascularization is an adaptive biological response to hypoxia/ischemia^35,36^. In murine oxygen-induced retinopathy model, hypoxic retina produces high levels of VEGFA, that results in abnormal outgrowth of new blood vessels that are characterized by bulging and the presence of anastomose-like structures leading to dilatation and leakiness^35,36^. In this regard, our findings demonstrate that oxygen-induced retinopathy triggers activation of PKCθ in hypoxic retina and its deletion prevents hypoxia-induced retinal EC proliferation, tip cell formation and neovascularization. Together, these observations infer that PKCθ plays a crucial role in VEGFA-induced retinal neovascularization. Other studies have also suggested a role for the involvement of PKC isoforms such as PKCβ2 in OIR-induced retinal EC proliferation and neovascularization^37^.

PKCθ has been reported to mediate activation of various transcriptional factors, including AP1^25^. A large body of evidence indicates that AP1 plays an important role in cell proliferation and differentiation^38^. In addition, several studies have reported the importance of AP1 in the regulation of VEGFA expression by cytokines^24,39–41^. However, little is known about the role of specific members of the Fos and Jun family of AP1 transcriptional factors in VEGFA-induced angiogenic effects. In the present study, we show that VEGFA induces JunB expression via a mechanism involving PKCθ in mediating HRMVEC migration, sprouting, and tube formation. Many studies have shown that JunB antagonizes c-Jun in the regulation of cell proliferation and transformation^42,43^. However, other studies have also reported that by homodimerization, JunB positively regulates cell proliferation, while heterodimers of cJun and JunB exert negative regulation^44^. Indeed, our observations support this possibility, as VEGFA-induced AP1-DNA complexes do not contain detectable c-Jun or JunD levels and VEGFA had little effect on the expression of the Fos family of AP1 transcriptional factors in HRMVECs. It should be noted that, consistent with our observations, other studies have also reported that JunB augments tumor angiogenesis and invasion^45–47^. Besides, the present observations show that hypoxia induces JunB expression in retina and its EC-specific knockdown inhibits hypoxia-induced EC proliferation, tip cell formation and neovascularization. Previously, we have shown that Fra1 and c-Jun mediate ischemia-induced angiogenesis^18^. These observations along with our findings that VEGFA and OIR induce c-Jun and JunD expression to some level, suggest that these protooncogenes might be involved in the mediation of other signaling events that may be required for angiogenesis.

The role of VEGFRs in endothelial cell sprouting and vessel branching is known^7,8^. It was reported that VEGFR2 and VEGFR3 heterodimers are essential for sprouting angiogenesis in mice and development of new blood vessels in zebrafish^7,8^. It was also reported that genetic deletion of VEGFR3 up-regulates VEGFR2 expression in vivo, constituting a compensatory feedback loop between VEGFR2 and VEGFR3^11,48^. Despite these studies emphasizing the importance of VEGFR2 and VEGFR3 interactions in developmental angiogenesis, nothing is known in regard to their crosstalk in pathological retinal angiogenesis. In this context, our studies provide the first evidence for a role of VEGFR3 in VEGFA/VEGFR2-induced pathological retinal angiogenesis. Specifically, our findings reveal that VEGFA induces VEGFR3 expression in HRMVECs and depletion of its levels blunts VEGFA-induced HRMVEC migration, sprouting and tube formation. These findings infer that VEGFR3 mediates VEGFA-induced angiogenic events in HRMVECs. Furthermore, suppression of VEGFR3 levels attenuates hypoxia-induced retinal EC proliferation, tip cell formation and neovascularization, suggesting a role for VEGFR3 in retinal neovascularizaion. As VEGFA stimulated VEGFR2 tyrosine phosphorylation and downregulation of VEGFR2 levels prevented VEGFA-induced VEGFR3 levels, it is likely that VEGFR2 activation is required for VEGFA-induced VEGFR3 expression. Previous studies have shown that downregulation of VEGFR3 levels leads to increased expression and activity of VEGFR2 in the modulation of vascular permeability^13^. On the other hand, a role for dimerization of VEGFR2 with VEGFR3 was reported in the regulation of sprouting angiogenesis^8^. In this context, our findings show that downregulation of VEGFR3 levels had no major effect on VEGFR2 levels and instead VEGFR2 activation is required for VEGFA-induced VEGFR3 expression, suggesting lack of a compensatory link between these receptors in the regulation of retinal neovascularization. Similarly, since VEGFR2 exists in complex with VEGFR3 under basal condition and in response to VEGFA they appear to be dissociated from each other, it may be suggested that heterodimerization of these receptors may not be needed for VEGFA/hypoxia-induced retinal neovascularization. Because VEGFA induces VEGFR3 expression and this effect depends on activation of VEGFR2 and downregulation of either receptor reduces retinal neovascularization, it can be assumed that VEGFR3 expression is required for VEGFA/VEGFR2-induced retinal angiogenesis. In addition, in exploring the potential mechanisms by which VEGFR3 plays a role in VEGFA/VEGFR2-induced angiogenic signaling, our results show that VEGFA stimulates STAT3 activation in a delayed manner and that this effect depends on the involvement of both VEGFR2 and VEGFR3. Since blockade of STAT3 attenuates VEGFA-induced HRMVEC migration, sprouting and tube formation and hypoxia-induced retinal neovascularization, it is likely that VEGFR2-mediated VEGFR3 expression is required for the delayed activation of STAT3 in the modulation of retinal neovascularization. A role for STAT3 in pathological retinal angiogenesis has also been reported previously^49^. It is interesting to note that depletion of VEGFR3 levels by its siRNA attenuates hypoxia-induced STAT3 phosphorylation, retinal EC proliferation, tip cell formation and neovascularization. In addition, soluble VEGFR3 protein while having little effect on filopodia formation also blunted retinal EC proliferation and neovascularization. These observations suggest that VEGFR3 mediates hypoxia-induced retinal neovascularization in a ligand dependent and independent manner. Although PKCθ-JunB-VEGFR3 signaling plays a role in VEGFA-induced migration, sprouting and tube formation of HRMVECs and hypoxia-induced retinal neovascularization, it appears that activation of this signaling while required for MRMVEC proliferation was not sufficient to trigger HRMVEC proliferation. This differential role of PKCθ-JunB-VEGFR3 signaling in human versus mouse retinal EC proliferation may be attributed to species differences. In fact, we have also demonstrated previously species specific involvement of LMCD1 in the regulation of aortic smooth muscle cell proliferation in human versus mice^50^.

In conclusion, our findings for the first time provide evidence for the involvement of PKCθ-JunB signaling in VEGFA/VEGFR2-induced expression of VEGFR3, targeting STAT3 activation in mediating OIR-induced retinal neovascularization. In view of these observations, it may be suggested that use of a combination of soluble VEGFR2 and VEGFR3 proteins might be a better therapeutic approach than the current ones that target only VEGFR2 to treat retinal neovascularization.

## Supplementary information

Supplementary Figure Legends

Supplementary Figure 1

Supplementary Figure 2

Supplementary Figure 3

Supplementary Figure 4

Supplementary Figure 5
