# Supplementary material for: PKCθ-JunB axis via upregulation of VEGFR3 expression mediates hypoxia-induced pathological retinal neovascularization
Source: Cell Death Dis. 2020 May 7;11(5):325. doi: 10.1038/s41419-020-2522-0 (PMC7206019; doi:10.1038/s41419-020-2522-0)
Supplement: Supplementary file 1 — Supplementary Figure Legends [file 41419_2020_2522_MOESM1_ESM.docx]

**PKCθ-JunB axis via upregulation of VEGFR3 expression mediates hypoxia-induced pathological retinal neovascularization**

Raj Kumar, Arul M Mani, Nikhlesh K Singh and Gadiparthi N. Rao

Department of Physiology, University of Tennessee Health Science Center, Memphis, TN 38163, USA

**Supplementary Information**

**Supplementary Figure Legends**

**Figure S1. Forced expression of JunB rescues VEGFA-induced migration, sprouting and tube formation in PKCθ knockout endothelial cells. A-C. Retinal ECs from PKCθ^-/-^ mice were transfected with empty vector or JunB expression vector and the rescue effects of JunB overexpression on migration (A**), sprouting (**B**) and tube formation (**C**) are shown. The bar graphs represent quantitative analysis of three independent experiments. The values are presented as Mean ± SD. * p < 0.01 vs vehicle; ** p < 0.01 vs VEGFA; ^#^ p < 0.01 vs pCMV vector. Scale bars in panels B and C are 50 μm and 200 μm, respectively.

**Figure S2. JunB binds VEGFR3 promoter in response to VEGFA.** **A.** Transfac analysis of ~0.8 kb VEGFR3 promoter showing potential transcriptional factor binding sites. **B.** Nuclear extracts of control and the indicated time periods of VEGFA (40 ng/ml)-treated HRMVECs were analyzed by EMSA using AP1-binding site at -543 nt in the VEGFR3 promoter as a biotin-labeled probe. **C.** Nuclear extracts of control and 2 hrs of VEGFA (40 ng/ml)-treated HRMVECs were analyzed by EMSA using WT and mutant AP1-binding element as biotin-labeled probes. **D.** Nuclear extracts of control and 2 hrs of VEGFA (40 ng/ml)-treated cells were analyzed by supershift EMSA using normal IgG, anti-JunB, anti-cJun or anti-JunD antibodies. **E.** HRMVECs were transfected with siControl or siPKC**θ (100 nM), quiesced,** treated with and without VEGFA (40 ng/ml) for 2 hrs and nuclear extracts were prepared and analyzed by EMSA for AP1 binding activity using AP1-binding site at -543 nt as a biotin-labeled probe.

**Figure S3. Administration of soluble VEGFR2 blocks retinal neovascularization. A.** Eyes from normoxic and 72 hrs of hypoxic mice pups that were injected intravitreally with vehicle or soluble VEGFR2 (0.05 μg/0.5 μl/eye) at P11, P12 and P13 were enucleated, fixed, sections were made and coimmunostained for CD31 and Ki67. The bar graph shows quantification of proliferating ECs per section. **B.** All the conditions were the same as in panel **A** except that eyes were enucleated at P17 (i.e., 120 hrs of hypoxia), fixed, retinas were isolated, stained with isolectin B4, flat mounts were made and examined for filopodia at 40X magnification (scale bar, 50 μm). Bar graph shows quantification of the number of filopodia/unit vessel length. **C**. All the conditions were the same as in panel **B** except that the flat mounts were examined for retinal vascularization. Retinal vascularization is shown in the first column at 2.5X magnification (scale bar, 500 μm). Neovascularization is highlighted in red in the second column. The third column shows the selected rectangular areas of the images in the first column at 10X magnification (scale bar, 200 μm). **D & E.** Retinal neovascularization (**D**) and avascular area (**E)** were determined as described in “Materials and Methods.” The values are presented as Mean ± SD. * p < 0.01 vs normoxia; ** p < 0.01 vs control hypoxia.

**Figure S4. STAT3 mediates VEGFA-induced angiogenic events in HRMVECs. A.** Upper panel: Western blot analysis of HRMVECs infected with Ad-GFP or Ad-dnSTAT3 (40 moi) for STAT3 overexpression. Lower panel: Cells were infected with Ad-GFP or Ad-dnSTAT3 (40 moi), quiesced and subjected to VEGFA (40 ng/ml)-induced cell migration. **B-D**. All the conditions were the same as in panel A except that after quiescence cells were subjected to VEGFA (40 ng/ml)-induced DNA synthesis (B) sprouting (C) and tube formation (D) assays. The values are presented as Mean ± SD. * p < 0.01 vs Ad-GFP alone; ** p < 0.01 vs Ad-GFP + VEGFA. Scale bars in panels C and D are 50 μm and 200 μm, respectively.

**Figure S5. Blockade of STAT3 activation inhibits retinal neovascularization. A.** Eyes from normoxic and 72 hrs of hypoxic mice pups that were injected intravitreally with Ad-GFP or Ad-dnSTAT3 (40 moi/0.5 μl/eye) at P11 were enucleated, fixed, sections were made and coimmunostained for CD31 and Ki67. The bar graph shows quantification of proliferating ECs per section. Western blot shows the overexpression of GFP and STAT3 with normalization for β-tubulin. **B.** All the conditions were the same as in panel **A** except that eyes were enucleated at P17 (i.e., 120 hrs of hypoxia), fixed, retinas were isolated, stained with isolectin B4, flat mounts were made and examined for filopodia at 40X magnification (scale bar, 50 μm). Bar graph shows quantification of the number of filopodia/unit vessel length. **C**. All the conditions were the same as in panel **B** except that the flat mounts were examined for retinal vascularization. Retinal vascularization is shown in the first column at 2.5X magnification (scale bar, 500 μm). Neovascularization is highlighted in red in the second column. The third column shows the selected rectangular areas of the images in the first column at 10X magnification (scale bar, 200 μm). **D & E.** Retinal neovascularization (**D**) and avascular area (**E)** were determined as described in “Materials and Methods.” The values are presented as Mean ± SD. * p < 0.01 vs normoxia; ** p < 0.01 vs AdGFP + hypoxia.
