# Supplementary figures and images for: PKCθ-JunB axis via upregulation of VEGFR3 expression mediates hypoxia-induced pathological retinal neovascularization
Source: Cell Death Dis. 2020 May 7;11(5):325. doi: 10.1038/s41419-020-2522-0 (PMC7206019; doi:10.1038/s41419-020-2522-0)

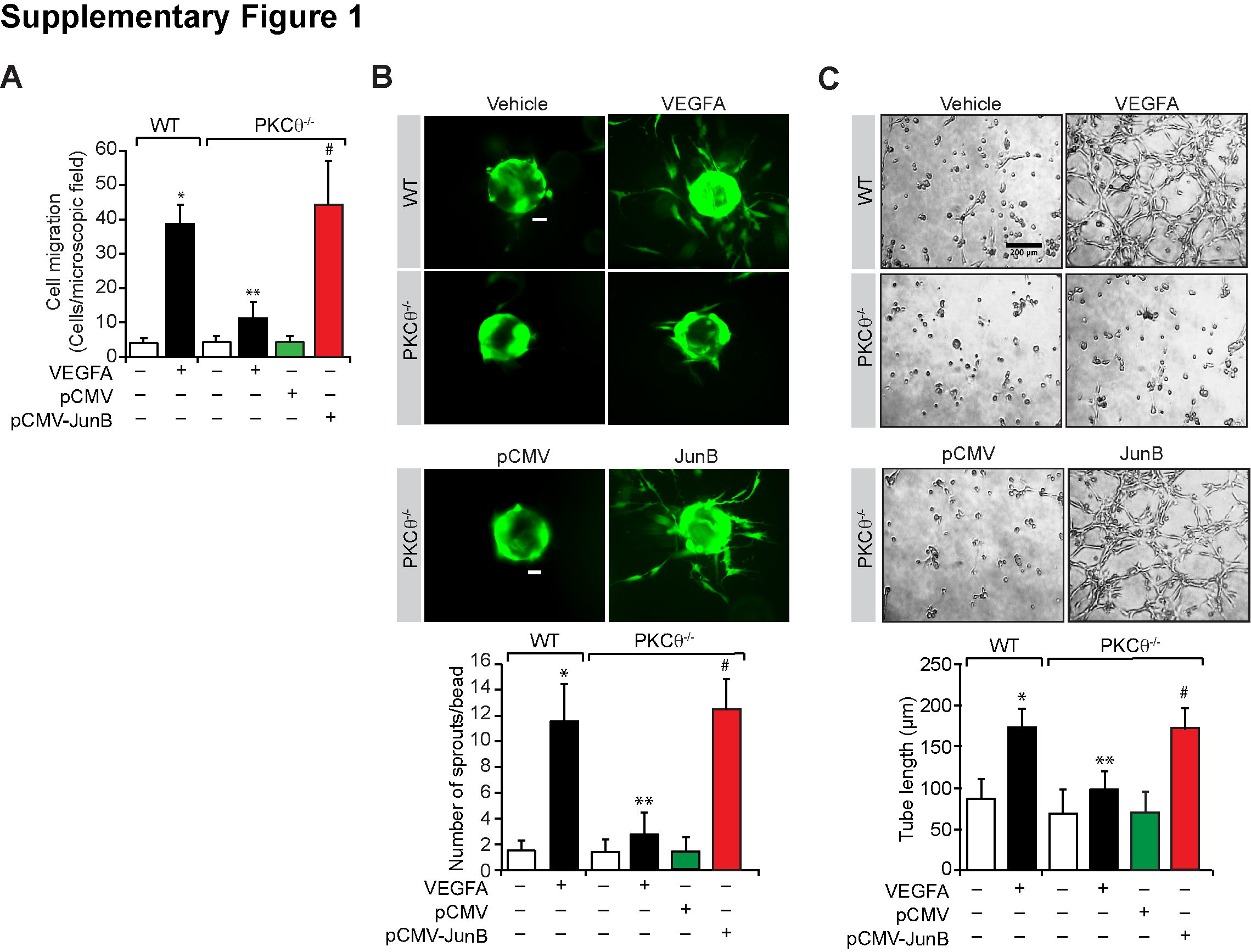

Supplement: Supplementary file 2 — Supplementary Figure 1 [file 41419_2020_2522_MOESM2_ESM.tif]

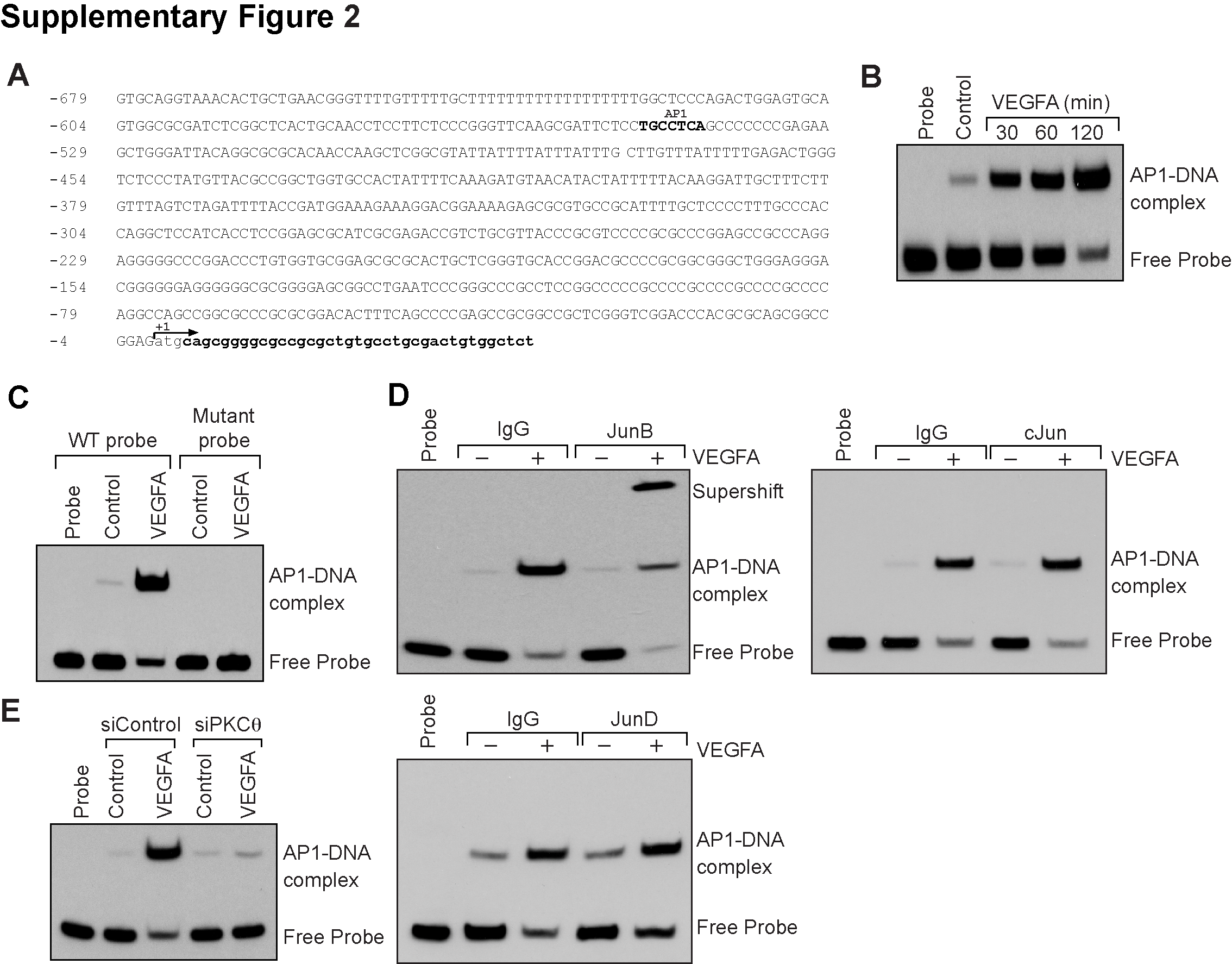

Supplement: Supplementary file 3 — Supplementary Figure 2 [file 41419_2020_2522_MOESM3_ESM.tif]

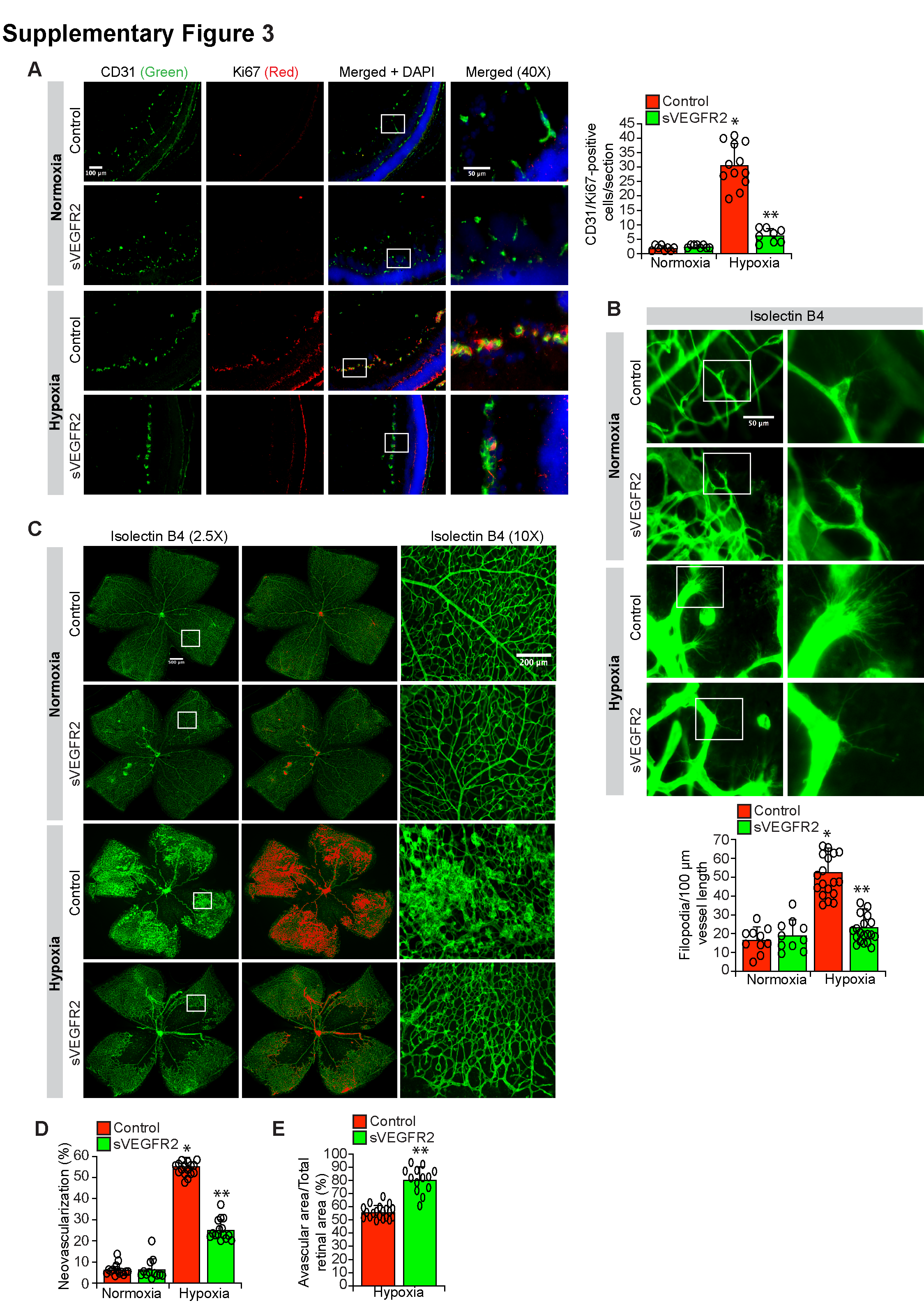

Supplement: Supplementary file 4 — Supplementary Figure 3 [file 41419_2020_2522_MOESM4_ESM.tif]

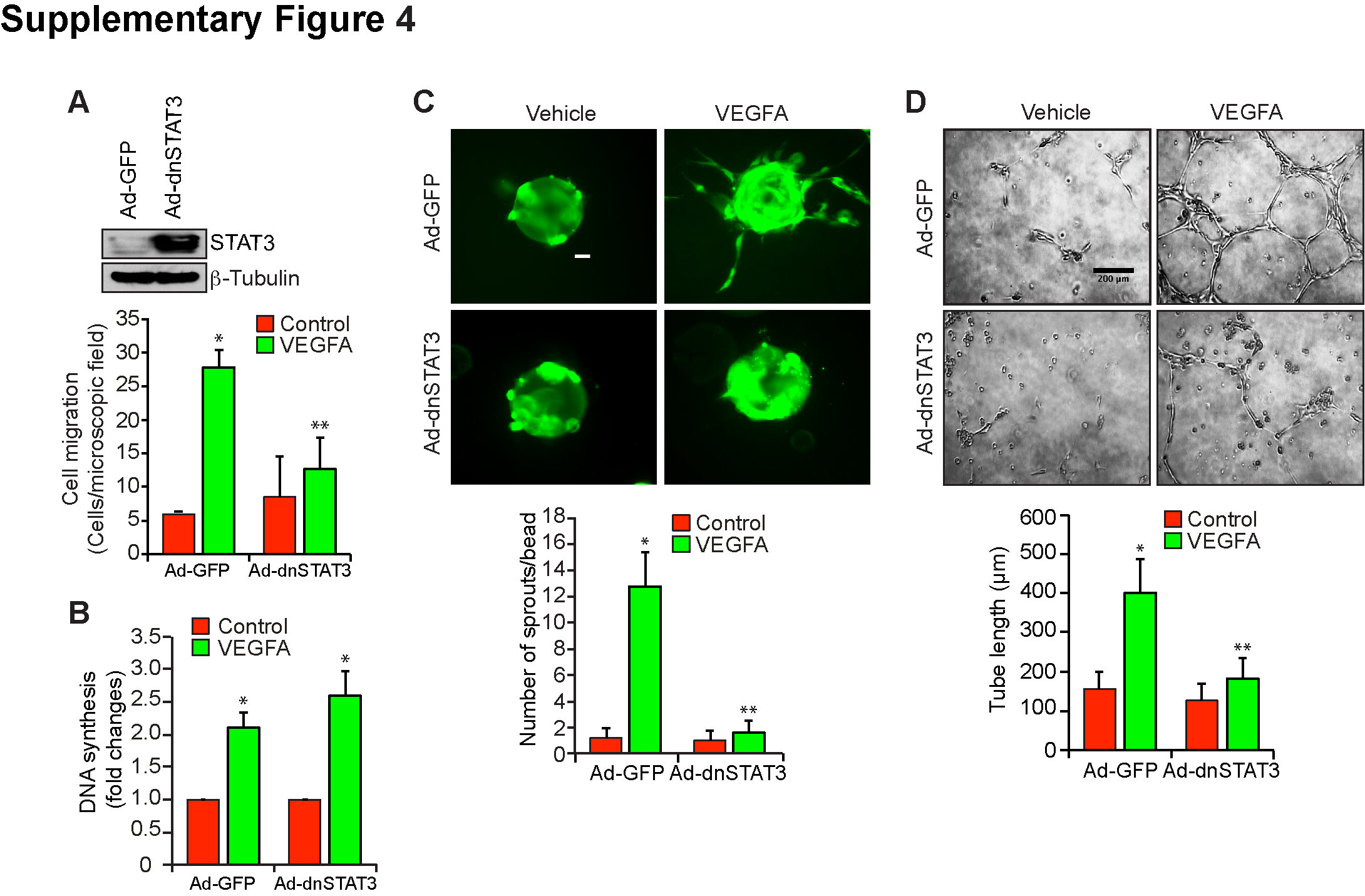

Supplement: Supplementary file 5 — Supplementary Figure 4 [file 41419_2020_2522_MOESM5_ESM.tif]

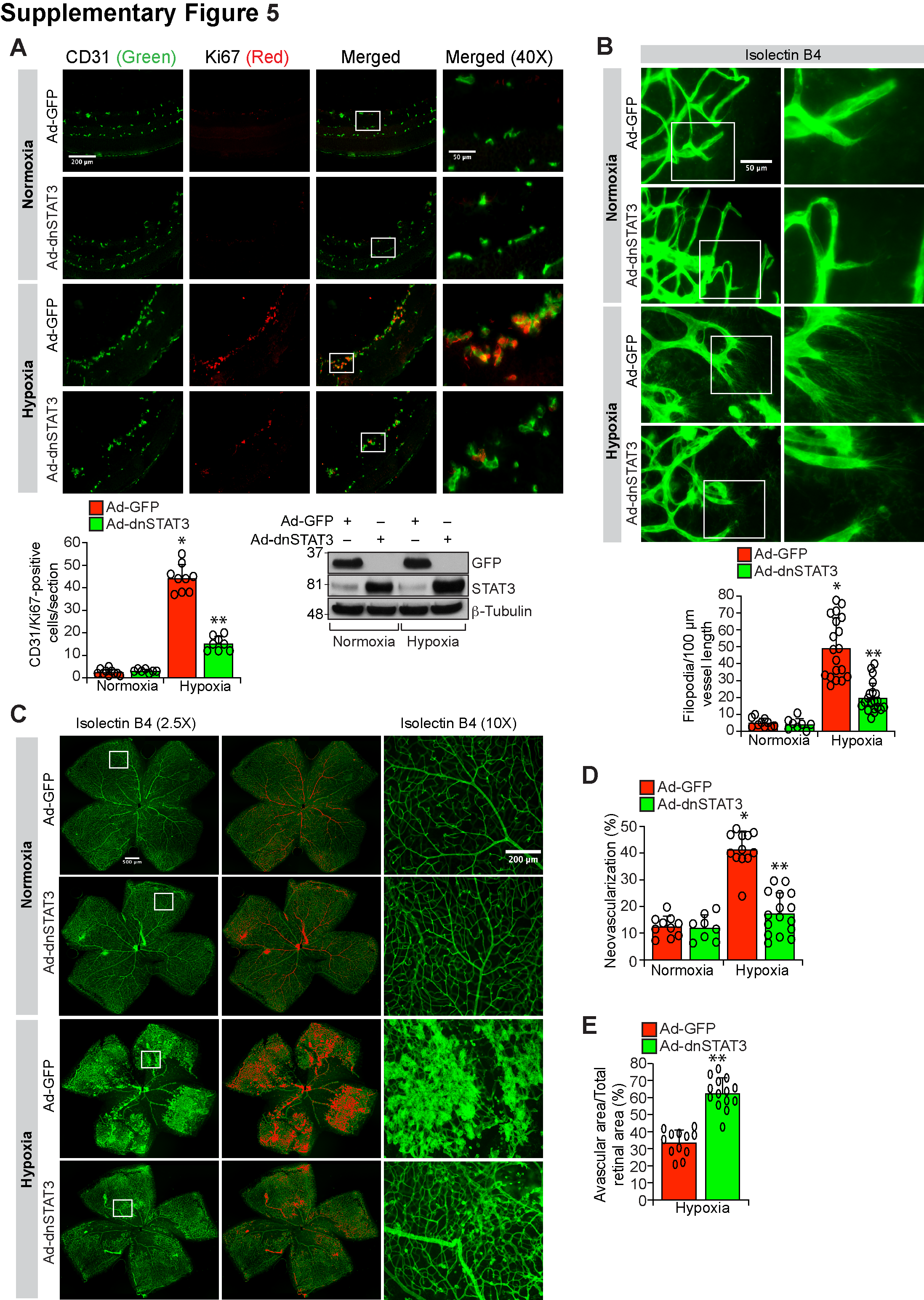

Supplement: Supplementary file 6 — Supplementary Figure 5 [file 41419_2020_2522_MOESM6_ESM.tif]
